# Impact of dual active ingredients long-lasting insecticidal nets on the genetic structure of insecticide resistant populations of *Anopheles gambiae* in Southern Benin

**DOI:** 10.1186/s12936-025-05308-7

**Published:** 2025-03-04

**Authors:** Boulais Yovogan, Armel Djènontin, Martin C. Akogbéto, Arthur Sovi, Constantin J. Adoha, Arsène Fassinou, Albert S. Salako, Esdras M. Odjo, Landry Assongba, Manfred Accrombessi, Edouard Dangbénon, Bénoît S. Assogba, Idelphonse Ahogni, Antoine A. Missihoun, Serge Akpodji, Fiacre Agossa, Roséric Azondékon, Come Zinsou Koukpo, Gil G. Padonou, Corine Ngufor, Jackie Cook, Natacha Protopopoff, Louisa A. Messenger, Clément Agbangla

**Affiliations:** 1 Faculté des Sciences et Techniques de l’Université d’Abomey-Calavi, Abomey-Calavi, Bénin; 2 Centre de Recherche Entomologique de Cotonou, Cotonou, Bénin; 3 Faculty of Infectious and Tropical Diseases, Department of Disease Control, London School of Hygiene and Tropical Medicine, London, UK; 4 Faculté d’Agronomie, Université de Parakou, Parakou, Bénin; 5 Disease Control and Elimination Theme (DCE), Medical Research Council Unit, The Gambia at the London School of Hygiene and Tropical Medicine (MRCG-LSHTM), Banjul, The Gambia; 6 Medical Research Council (MRC) International Statistics and Epidemiology, Epidemiology Group, London School of Hygiene and Tropical Medicine, London, UK; 7 Department of Environmental and Occupational Health, School of Public Health, University of Nevada, Las Vegas, NV USA

## Abstract

**Background:**

Insecticide resistance amongst vector populations is a major challenge, exacerbated by the continued use of the same active ingredients. The present study assessed the impact of long-lasting insecticidal nets (LLINs) bi-treated with chlorfenapyr-alphacypermethrin (PY-CFP LLIN) or pyriproxyfen-alphacypermethrin (PY-PPF LLIN) on the genetic structure of resistant populations of *Anopheles gambiae* in 60 clusters divided into three arms from three districts in southern Benin.

**Methods:**

The study was conducted between September 2019 and October 2021 in 123 villages grouped in 60 clusters. Mosquitoes were collected indoors and outdoors using human landing catches (HLCs) in 4 households in each cluster every 3 months. After morphological identification, a subsample of *An. gambiae *sensu lato (*s.l.)* was analysed by PCR to detect the molecular species and the presence of L1014F *vgsc-kdr* and G119S*-ace-1* mutations.

**Results:**

*Anopheles coluzzii* (56.9%) and *An. gambiae *sensu stricto (*s.s*.) (42.8%), with a few hybrids (0.2%), were identified within 4242 samples of *An. gambiae* tested. The frequency of L1014F *vgsc-kdr* decreased in *An. coluzzii* collected both indoors and outdoors locations in the PY-CFP LLIN and PY-PPF LLIN arms post-intervention compared to baseline. In *An. gambiae*, the frequency of the L1014F allele decreased in year one but increased above baseline in year 2. In both species, the allelic frequency of G119S*-ace-1* was < 10%. For L1014F *vgsc-kdr*, the fixation index was positive (*F*_*IS*_ > 0) in both species. However, it was negative (*F*_*IS*_ < 0) for the presence of G119S*-ace-1*. Weak genetic differentiation, especially in the PY-PPF LLIN and PY-CFP LLIN arms (*F*_*ST*_ ≤ 0.05), was observed in *An. gambiae s.s.* populations with L1014F *vgsc-kdr*, while it was generally higher for both species with G119S*-ace-1*.

**Conclusion:**

The frequency of the L1014F *vgsc-kdr* resistance allele was high, while that of the G119S-*ace-1* allele was low throughout the study period. Consistent changes in allele frequencies were not observed in any of the treatment arms suggesting that the pyrethroid component of dual AI (active ingredients) nets continues to select for the resistant allele and there is little if any evidence that the non-pyrethroid insecticide selects for the wild-type *kdr* allele.

**Supplementary Information:**

The online version contains supplementary material available at 10.1186/s12936-025-05308-7.

## Background

Global progress in the fight against malaria has remained stable in recent years due to increased resistance of mosquitoes to insecticides and parasites to treatments, as well as insufficient funding for programmes to combat the disease [1]. Most insecticides used in public health vector control are neurotoxic to mosquitoes [2]. Pyrethroids and organochlorines target receptors in the axons of neurons, while carbamates and organophosphates interfere with acetylcholinesterase, an enzyme involved in synaptic transmission [3, 4]. Studies conducted in Benin have confirmed widespread vector resistance to pyrethroids (alpha-cypermethrin, permethrin and deltamethrin) used to treat long-lasting insecticidal nets (LLINs) [5–7]. As a result, the effectiveness of LLINs in areas of high vector resistance has been declining [8].

Resistance by modification of the insecticide target site has been observed in *Anopheles gambiae *sensu lato (*s.l*.), mediated by genetic mutations (*vgsc-*1014F, *vgsc-*1570Y and *vgsc-*402L) in voltage-gated sodium channels (*vgsc*) and *ace1-*119S mutations in the acetylcholinesterase gene (*ace-1*) [9, 10]. In addition, resistance can be mediated by metabolic mechanisms, which result in increased activity levels of enzymes involved in insecticide degradation [11]. In Benin, these different resistance mechanisms have been reported in several agro-ecological zones [12–15].

In view of this situation, new molecules with different modes of action are essential to overcome insecticide resistance mechanisms in mosquitoes [16]. A first attempt to manage insecticide resistance caused by nets was the development of insecticide-treated nets (ITNs) that incorporated a pyrethroid and a synergist, piperonyl butoxide (PBO), which has been shown to increase vector mortality with resistance involving overexpression of mono-oxygenases [17, 18]. However, the effectiveness of these ITNs depends on the extent to which mono-oxygenase enzymes are driving resistance in vector populations [19]. In Benin, the addition of the synergist PBO did not fully restore the sensitivity of vectors to pyrethroids in certain localities [20]. Recent research exploring other insecticide classes has identified bi-treated LLINs as a promising option for vector control [21–23]. In addition to pyrethroids, these nets are treated with either pyriproxyfen or chlorfenapyr. Chlorfenapyr, a pyrrole, acts by disrupting ATP (adenosine triphosphate) production in the mitochondria via its oxidizing compound (tralopyril: CL303628). This disruption of oxidative phosphorylation leads to the insect’s death [24]. Pyriproxyfen is a growth regulator, a juvenile hormone analogue known to disrupt female reproduction and egg fertility, as well as larval development in insects [25, 26].

Given the mode of action of the new ingredients (chlorfenapyr and pyriproxyfen) contained in these LLINs, they may exert resistance selection pressure in vectors. For example, in Mali, Norris et al*.* [27] showed an increase in the frequency of the L1014F *vgsc-kdr* gene in *Anopheles coluzzii* after intensive use of LLINs. In certain regions of Benin where the insecticide pirimiphos-methyl was used for indoor residual spraying (IRS), putative resistance was subsequently observed, leading to its replacement by the insecticide clothianidin [28].

For prospective insecticide resistance management strategies to succeed, there needs to be a clear understanding of the specificity of resistance mechanisms to individual insecticides, the likelihood of selecting for cross-resistance mechanisms and the impact of intervention deployment on population gene flow and genetic diversity. In 2020, Interceptor G2 (pyrethroid-chlorfenapyr LLIN) and Royal Guard (pyrethroid- pyriproxyfen LLIN) were distributed in Benin to protect populations in the Cove-Ouinhi-Zangnanado (CoZO) health zone as part of a cluster randomised controlled trial (RCT) in Southern Benin. The present study aimed to evaluate the impact of these nets on the genetic structure of insecticide resistant populations of *An. gambiae s.l.*

## Methods

### Study area

**Fig. 1:**
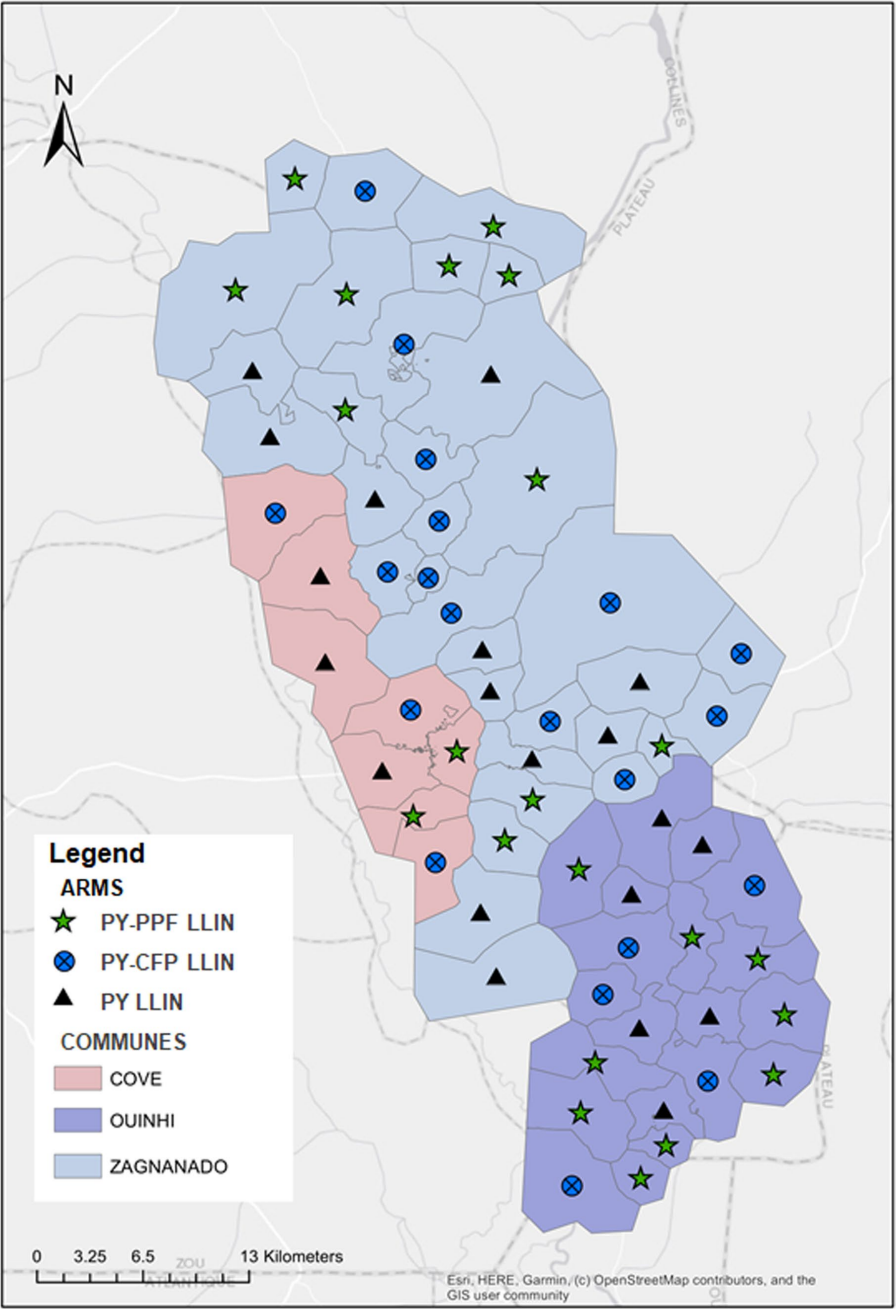
Map of the study area. PY LLIN: standard LLIN, LLIN treated with pyrethroid only; PY-CFP LLIN: LLIN bi-treated with pyrethroid-chlorfenapyr; PY-PPF LLIN: LLIN bi-treated with pyrethroid-pyriproxyfen

The study was nested in a RCT that was carried out in the CoZO health zone, Zou department of Southern Benin [29]. In this region, the malaria prevalence is very high, with a peak of cases between May and October [30]. The main income-generating activities were agriculture, trade, fishing and hunting. LLINs, which are distributed nationwide every 3 years, are the main means of protection against mosquito bites in the region. The CoZO health zone comprises 123 villages with a population of around 220,000. It was grouped into 60 clusters assigned to three study arms: Interceptor LLINs (LLINs treated with pyrethroid only; control arm; PY LLINs), Interceptor G2 (LLINs bi-treated with pyrethroid-chlorfenapyr; PY-CFP LLINs) and Royal Guard (LLINs bi-treated with pyrethroid-pyriproxyfen; PY-PPF LLINs). Each cluster (Fig. 1) comprised an average of 200 households for 1200 residents.

### Mosquito collection and morphological identification

Adult mosquitoes were collected in all clusters over three collection rounds [from September to October in 2019 (baseline), September–October 2020 and September–October 2021 (post-LLIN distribution)], using human landing catches (HLCs), i.e. three collections per arm (20 clusters/arm). In each cluster, four houses located approximately 15–20 m apart were selected from the survey census database organized in 2019. In each house, two collectors (1 inside and 1 outside) were used from 7 pm to 1am, and two others from 1 to 7am. A total of 2880 collectors were used in 720 households in this study. *Anopheles* mosquitoes collected were identified morphologically using the taxonomic identification key of Coetzee [31].

### Molecular analyses

A subsample of the *An. gambiae* complex collected indoors and outdoors was randomly selected. The heads and thoraxes of each of the *An. gambiae* complex were used to detect infection with *Plasmodium falciparum* sporozoites by ELISA-CSP [32]. Their abdomens, legs and wings were used for species identification using the PCR protocol of Santolamazza et al*.* [33]. The genotypes of the L1014F *vgsc-kdr* and G119S*-ace-1* mutations were determined in species of the *An. gambiae* complex following the protocols of Martinez-Torres et al*.* [11]; and Weill et al. [34], respectively.

### Statistical analysis

Data were entered twice into databases designed with CS Pro 7.2 software and analysed with Stata 15.0 (Stata Corp., College Station, TX). The genetic make-up of the *An. gambiae* complex was determined by calculating the allelic frequencies of the L1014F *vgsc-kdr* and G119S*-ace-1* mutations. The proportion of each allelic frequency was obtained using the binomial test function in R software version 4.3.2. A Chi-square test was used to assess the difference in the frequencies of infection between resistant and susceptible alleles. The level of significance was set at 0.05.

In the genetic analyses, sub-populations were assigned according to the different types of nets distributed (study arm). These included the PY LLIN sub-population, where standard nets were distributed, the PY-PPF-LLIN sub-population, where pyriproxyfen-incorporated nets were distributed, and the PY-CFP-LLIN sub-population, where chlorfenapyr-incorporated nets were distributed. Panmixia within *An. gambiae* complex populations in the different study arms was verified using the Hardy–Weinberg equilibrium (HWE) test. Indices of observed heterozygosity (*Ho*), expected heterozygosity (*He*), fixation index (*F*_*IS*_) and genetic differentiation (*F*_*ST*_) within *An. gambiae* populations were calculated according to the formulas of Weir and Cockerham [35] and Robertson and Hill [36], integrated into Genepop software version 8.4.2. The fixation index (*F*_*IS*_) was used to quantify divergence from panmixia, where a *F*_*IS*_ value < 0 indicates an excess of heterozygosity, while *F*_*IS*_ > 0 indicates a deficit of heterozygosity. The variation in *F*_*IS*_ ranges from −1, then all loci are heterozygous for the same alleles, to + 1 if all loci are homozygous for different alleles. Similarly, *F*_*IS*_ = 0 means that allele frequencies conform to the expectations of HWE. The criteria defined by Hartl et al*.* [37] were used to assess genetic differentiation within populations, classifying it as weak (*F*_*ST*_ ≤ 0.05), moderate ([0.05–0.15]), significant ([0.15–0.25]), or highly significant (*F*_*ST*_ > 0.25). These parameters were compared before and after the nets were deployed in the different study arms.

## Results

### *Anopheles* species composition

**Fig. 2:**
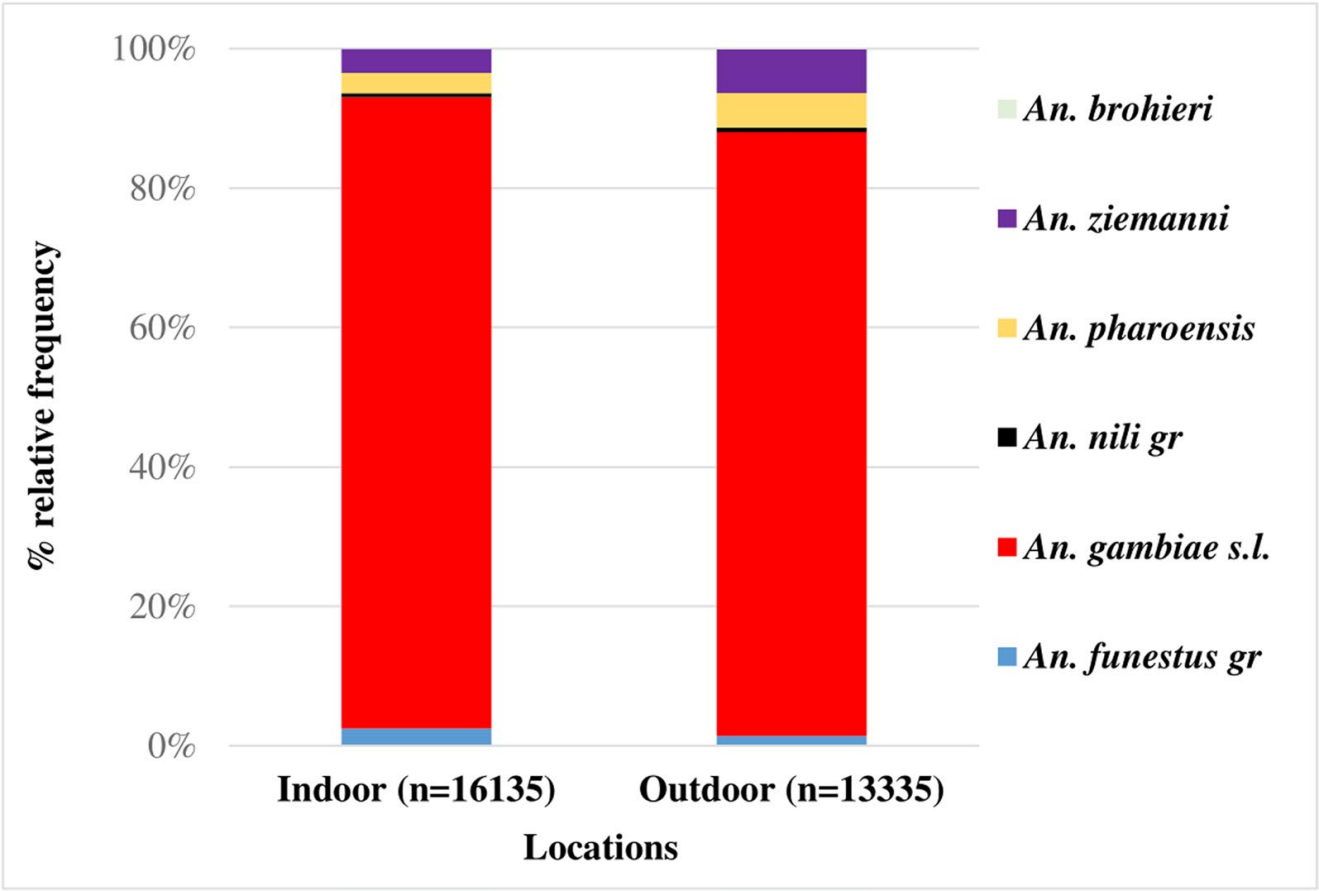
*Anopheles* species composition

A total of 29,470 mosquitoes belonging to six different anopheline complexes were collected in the study area. The *An. gambiae* complex accounted for 88.9% of the total *Anopheles* collected. There were significantly higher proportions of *An. gambiae* indoors (55.9%, n = 14,617, 95% CI: 55.2–56.5) versus outside (44.1%, n = 11,549, 95% CI: 43.5–44.7); p < 0.0001. A similar trend was observed for *Anopheles funestus* [68.2% (n = 396, 95% CI: 64.2–71.9) indoors versus 31.8% (n = 185, 95% CI: 28.1–35.8) outdoors; p < 0.0001]; although this group was collected at comparatively low proportions. Other *Anopheles* species, namely *Anopheles ziemanni*, *Anopheles pharoensis*, *Anopheles nili* and *Anopheles brohieri,* were also found in low proportions (≤ 4%) both indoors and outdoors (Fig. 2).

### Allelic frequency of the L1014F *vgsc-kdr* mutation in *An. coluzzii* and *An. gambiae *sensu stricto

Of the 4242 *An. gambiae s.l.* specimens subjected to molecular analysis, two species, *An. coluzzii* (56.9%, n = 2423) and *An. gambiae *sensu stricto (*s.s*.) (42.8%, n = 1819), and a few hybrids (0.2%, n = 10) (*An. gambiae/coluzzii*) were identified. Indoors, *An. coluzzii* predominated (55.9%, n = 1434, CI 95%: 54.0–57.9) over *An. gambiae s.s.* (44.1%, n = 1128, CI 95%: 42.1–45.9), p < 0.0001). The same trends were observed outdoors.

Of a total of 1,797 specimens analysed during the baseline, 968 were *An. coluzzii*, distributed as follows: 34% (n = 333) in the PY LLIN arm, 35% (n = 341) in the PY-PPF LLIN arm and 31% (n = 294) in the PY-CFP LLIN arm. The remaining 829 specimens were *An. gambiae s.s*., distributed as follows: 32% (n = 267) in the PY LLIN arm, 31% (n = 252) in the PY-PPF LLIN arm and 37% (n = 310) in the PY-CFP LLIN arm. In the post-intervention (Post1 and Post2), a total of 2,443 specimens were analysed. Of these, 1,455 were *An. coluzzii*, distributed as follows: 34% (n = 500) in the PY LLIN arm, 33% (n = 484) in the PY-PPF LLIN arm and 33% (n = 471) in the PY-CFP LLIN arm. The remaining 988 specimens were *An. gambiae s.s.*, with 35% (n = 343) in the PY LLIN arm, 27% (n = 269) in the PY-PPF LLIN arm and 38% (n = 376) in the PY-CFP LLIN arm.

**Fig. 3:**
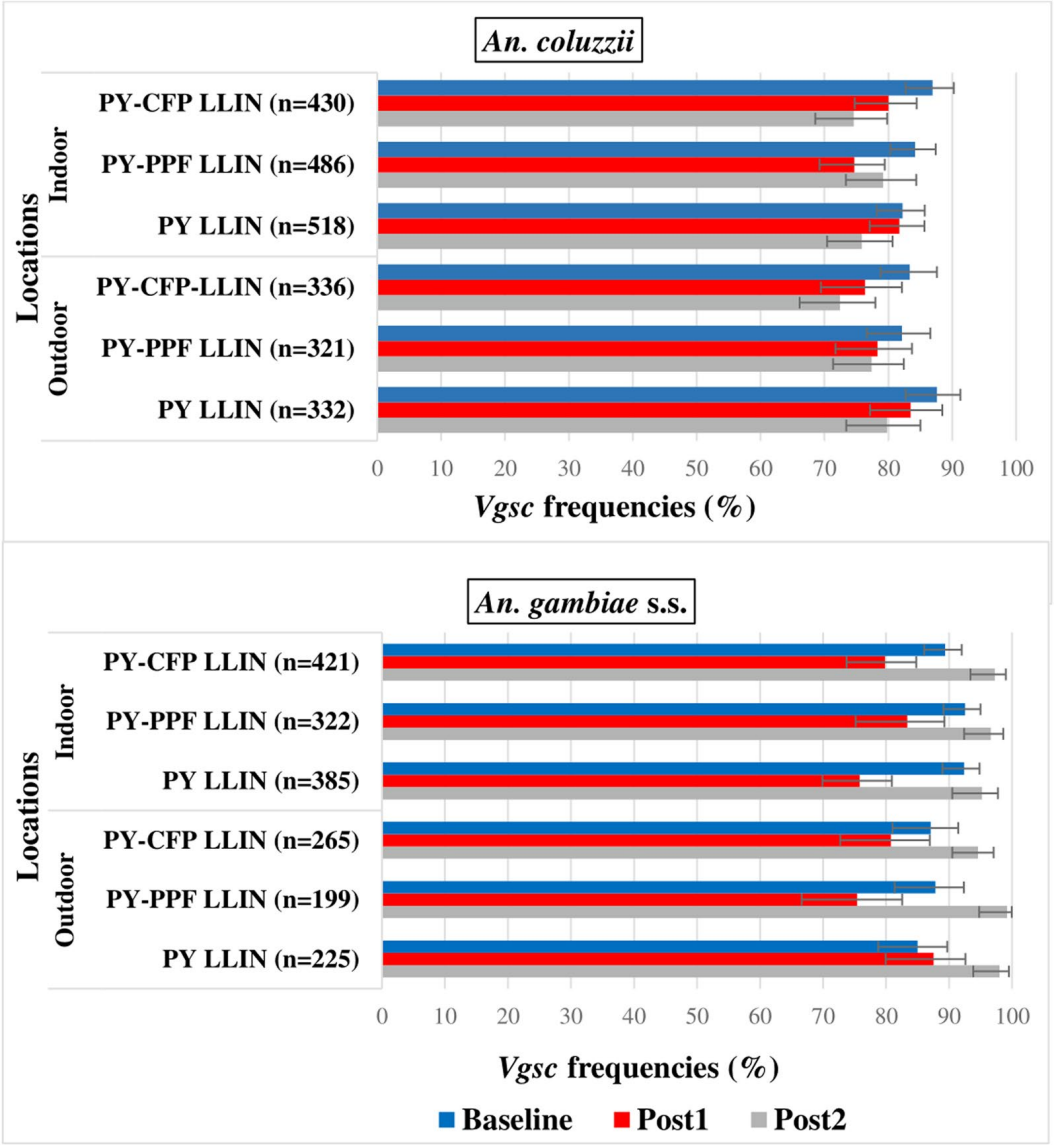
Allele frequencies of the L1014F *vgsc-kdr* mutation in species of the *An. gambiae* complex. PY LLIN: standard LLIN, LLIN treated with pyrethroid only; PY-CFP LLINs: LLIN bi-treated with pyrethroid-chlorfenapyr; PY-PPF LLIN: LLIN bi-treated with pyrethroid-pyriproxyfen; Post1: 1st year post-intervention; Post2: 2nd year post-intervention

In *An. coluzzii*, the frequency of the L1014F *vgsc-kdr* allelic indoors and outdoors after distribution of study LLINs was lower compared with baseline although none of the comparisons were statistically significant (Fig. 3). After distribution of study LLINs, the L1014F *vgsc-kdr* allelic frequency ranged from 74.6% (95% CI 68.6–79.8) in the PY-CFP LLIN arm to 81.7% (95% CI 77.1–85.6) in PY LLIN arm indoors and 72.4% (95% CI 66.1–77.9) in the PY-CFP LLIN arm to 83.5% (95% CI 77.1–88. 4) in the PY LLIN arm outdoors; at baseline the L1014F *vgsc-kdr* allelic frequency ranged from 82.2% (95% CI 78.2–85.7) in the PY LLIN arm to 86.9% (95% CI 82.7–90.2) in the PY-CFP LLIN arm indoors; and from 82.1% (95% CI 76.6–86.6) in the PY-PPF LLIN arm to 87.6% (95% CI 82.7–91.3) in the PY LLIN arm outdoors.

In *An. gambiae s.s*., there was a decrease in L1014F *vgsc-kdr* frequencies compared to baseline (Fig. 3) in the first post-intervention year. In contrast, an increase in L1014F *vgsc-kdr* frequencies were detected indoors and outdoors during the second post-intervention year in all arms compared with baseline (Fig. 3).

### Allele frequency of the G119S*-Ace-*1 mutation in *An. coluzzii* and *An. gambiae s.s.*

**Fig. 4:**
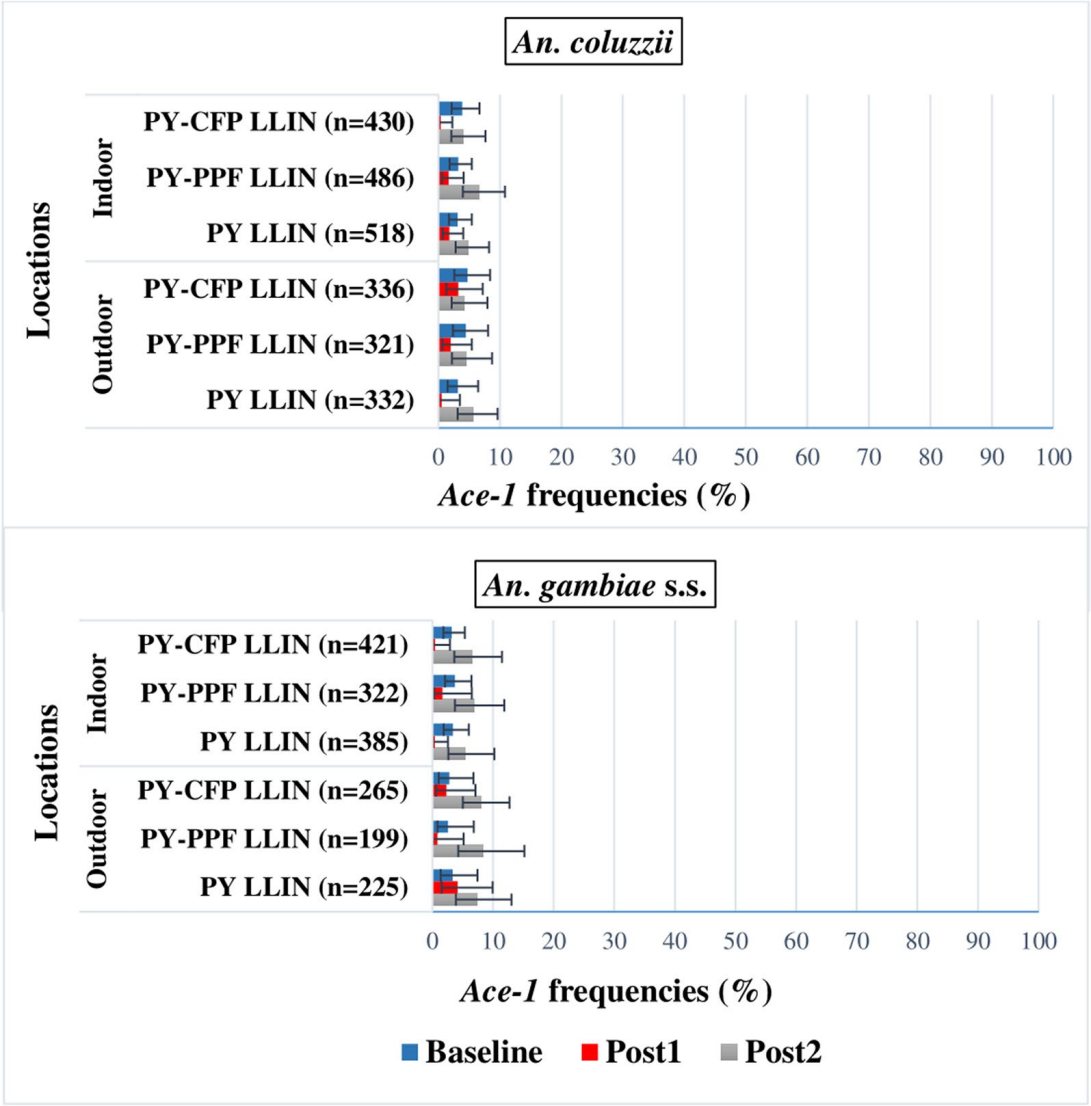
Allele frequency of the G119S*-ace-1* mutation in species of the *An. gambiae* complex. PY LLIN: standard LLIN, LLIN treated with pyrethroid only; PY-CFP LLINs: LLIN bi-treated with pyrethroid-chlorfenapyr; PY-PPF LLIN: LLIN bi-treated with pyrethroid-pyriproxyfen; Post1: 1st year post-intervention; Post2: 2nd year post-intervention

G119S*-ace-1* allele frequencies were generally low in all three study arms, whether baseline or post-intervention (Fig. 4), ranging from 0.36% (95% CI 0.02–2.57) to 8.33% (95% CI 4.29–15.17). In *An. coluzzii*, despite the generally low frequency, a decrease in G119S*-Ace-1* allele frequency was observed both indoors and outdoors after one year after distribution of LLINs in all study arms compared with baseline. But two years after LLIN distribution, there was a general increase in frequency. These variations were not significantly different (p > 0.05). Similar trends were obtained for *An. gambiae s.s* with a decrease in the first year then an increase in the second year post-intervention compared to baseline in all study arms (Fig. 4).

### Genotypic and allele frequency of L1014F *vgsc-kdr* and HWE deviations of *An. gambiae* *s.s.* and *An. coluzzii* populations

Resistant allele frequencies were very high (over 74%) at baseline and post-intervention in the different study arms. Indoors, within the L1014F *vgsc-kdr* locus, the frequency of homozygous resistant (RR) individuals was predominant in both species, with the highest peak observed at two years post-intervention in *An. gambiae s.s.* from the PY-CFP LLIN arm (95.6%). Heterozygous resistant (RS) individuals were present at moderate frequencies, especially in *An. coluzzii* in the different arms. Homozygous susceptible individuals (SS) were found at very low frequencies (Table 1). Significant deviations from HWE (p < 0.05), were observed in *An. coluzzii* populations (both baseline and post-intervention) except in the PY-PPF LLIN arm at two years post-intervention. However, fewer *An. gambiae s.s.* exhibited significant deviations from HWE except at one-year post-intervention in all arms and in the PY-CFP LLIN arm at baseline. (Table 1).

**Table 1:** Genotypic and allelic frequencies of L1014F *vgsc-kdr* and HWE deviations of indoor collected *An. gambiae* s.s and *An. coluzzii* populations

Species	Study arms		N *An*	Indoor			Fr (L1014F)	P-value HWE
				Genotypic frequencies				
				RR (%)	RS (%)	SS (%)		
*An. coluzzii*		Baseline	208	145 (69.7)	52 (25.0)	11 (7.6)	82.2	0.0341
	PY LLIN	Post1	167	117 (70.1)	39 (23.4)	11 (9.4)	81.7	0.0089
		Post2	143	88 (61.5)	41 (28.7)	14 (15.9)	75.9	0.0087
		Baseline	218	158 (72.5)	51 (23.4)	9 (5.7)	84.2	0.043
	PY-PPF LLIN	Post1	148	92 (62.2)	37 (25.0)	19 (20.7)	74.7	0.0004
		Post2	120	76 (63.3)	38 (31.7)	6 (7.9)	79.2	0.3604
		Baseline	168	130 (77.4)	32 (19.1)	6 (4.6)	86.9	0.0395
	PY-CFP LLIN	Post1	140	96 (68.6)	32 (22.9)	12 (12.5)	80.0	0.0015
		Post2	122	74 (60.7)	34 (27.9)	14 (18.9)	74.6	0.0039
*An. gambiae* s.s		Baseline	177	152 (85.9)	23 (13.0)	2 (1.3)	92.4	0.2635
	PY LLIN	Post1	124	78 (62.9)	32 (25.8)	14 (17.9)	75.8	0.0016
		Post2	84	77 (91.7)	6 (7.1)	1 (1.3)	95.2	0.1588
		Baseline	174	148 (85.1)	26 (14.9)	0 (0)	92.5	1
	PY-PPF LLIN	Post1	60	44 (73.3)	12 (20.0)	4 (9.1)	83.3	0.0408
		Post2	88	82 (93.2)	6 (6.8)	0 (0)	96.6	1
		Baseline	221	180 (81.5)	35 (15.8)	6 (3.3)	89.4	0.0231
	PY-CFP LLIN	Post1	109	74 (67.9)	26 (23.9)	9 (12.2)	79.8	0.0095
		Post2	91	87 (95.6)	3 (3.3)	1 (1.2)	97.3	0.0554

*An.* = *Anopheles gambiae* s.l.; N: number tested; PY LLIN: standard LLIN, LLIN treated with pyrethroid only; PY-CFP LLIN: LLIN bi-treated with pyrethroid-chlorfenapyr; PY-PPF LLIN: LLIN bi-treated with pyrethroid-pyriproxyfen; RR: homozygous resistant; RS: heterozygous resistant; SS: homozygous susceptible; Fr (L1014F) frequency of resistance allele; P value (HWE): P value for Hardy–Weinberg Equilibrium; Post1 = 1st year post-intervention; Post2 = 2nd year post-intervention

Similar trends were obtained in outdoor vector populations (*An. coluzzii* and *An. gambiae s.s.*) at baseline and post-intervention in the three arms (Supplementary file, Table S1). Indoors and outdoors, the frequency of L1014F *vgsc-kdr* was similar between the intervention arms (PY-CFP LLIN arm and PY-PPF LLIN arms) and the PY-LLIN arm in both *An. coluzzii* and *An. gambiae s.s.* (Supplementary file, Table S2).

### Genotypic and allelic frequencies of G119S-*ace-1* and HWE deviations of *An. gambiae* *s.s*. and *An. coluzzii* populations

Allelic frequencies of the resistant G119S-*Ace-1* mutation were very low in the three different arms study at baseline and post-intervention. Indoor, within the G119S *ace-1* locus, the frequencies of homozygous susceptible individuals (SS) were much higher in both species. Resistant heterozygous (RS) individuals were less represented, especially at the first-year post-intervention (ranging from 0.7% to 3.6%). No homozygous resistant individuals were observed at any time point (Table 2). Similar trends were obtained outside (Supplementary file, Table S3). There were no significant deviations from HWE in any population, except in *An. coluzzii* from the PY-CFP LLIN arm at the first-year post-intervention (Table 2). Indoors, no significant difference was observed between the frequency of G119S-*Ace-1* of the intervention arms (PY-CFP LLIN arm and PY-PPF LLIN arms) and that of the PY-LLIN arm in both *An. coluzzii* and *An. gambiae s.s*. The same trend was observed outdoors (Supplementary file, Table S4).

**Table 2:** Genotypic and allelic frequencies of G119S-*Ace-1* and HWE test indoor of *An. gambiae* s.s. and *An. coluzzii* populations

Species	Study arms		N *An.*	Indoor			Fr (G119S)	P-value (HWE)
				Genotypic frequencies				
				RR (%)	RS (%)	SS (%)		
*An. coluzzii*		Baseline	208	0 (0)	13 (6.3)	195 (93.8)	3.1	1
	PY LLIN	Post1	167	0 (0)	6 (3.6)	161 (96.4)	1.8	1
		Post2	143	0 (0)	14 (9.8)	129 (90.2)	4.9	1
		Baseline	218	0 (0)	14 (6.4)	204 (93.6)	3.2	
	PY-PPF-LLIN	Post1	148	0 (0)	5 (3.4)	143 (96.6)	1.7	1
		Post2	120	0 (0)	16 (13.3)	104 (86.7)	6.7	1
		Baseline	168	0 (0)	13 (7.7)	155 (92.3)	3.9	1
	PY-CFP-LLIN	Post1	140	0 (0)	1 (0.7)	139 (99.3)	0.4	< 0.0001
		Post2	122	0 (0)	10 (8.2)	112 (91.8)	4.1	1
*An. gambiae* s.s		Baseline	177	0 (0)	12 (6.8)	165 (93.2)	3.4	1
	PY LLIN	Post1	124	0 (0)	1 (0.8)	123 (99.2)	0.4	–
		Post2	84	0 (0)	9 (10.7)	75 (89.3)	5.4	1
		Baseline	174	0 (0)	13 (7.5)	161 (92.5)	3.7	
	PY-PPF-LLIN	Post1	60	0 (0)	2 (3.3)	58 (96.7)	1.7	1
		Post2	88	0 (0)	12 (13.6)	76 (86.4)	6.82	1
		Baseline	221	0 (0)	14 (6.3)	207 (93.7)	3.2	1
	PY-CFP-LLIN	Post1	109	0 (0)	1 (0.9)	108 (99.1)	0.5	–
		Post2	91	0 (0)	12 (13.2)	79 (86.8)	6.6	1

*An.* = *Anopheles gambiae* s.l.; N: number tested; PY LLIN: standard LLIN, LLIN treated with pyrethroid only; PY-CFP LLIN: LLIN bi-treated with pyrethroid-chlorfenapyr; PY-PPF LLIN: LLIN bi-treated with pyrethroid-pyriproxyfen; RR: homozygous resistant; RS: heterozygous resistant; SS: homozygous susceptible; Fr (R) frequency of resistance allele; P value (HWE): P value for Hardy–Weinberg Equilibrium; Post1 = 1st year post-intervention; Post2 = 2nd year post-intervention

### Fixation index in *An. gambiae s.s*. and *An. coluzzii*

Table 3 shows the fixation index (*F*_*IS*_) in *An. gambiae s.s* and *An. coluzzii* populations for L1014F-*vgsc-kdr* and G119S*-ace-1* mutations in different study arms indoors and outside dwellings.

**Table 3:** Fixation index (*F*_*IS*_) in *An. gambiae* s.s. and *An. coluzzii*

Locations/MIILDs	Periods	*F*_*IS*_ of Locus L1014F		*F*_*IS*_ of Locus G119S	
		*An. coluzzii*	*An. gambiae s.s*	*An. coluzzii*	*An. gambiae s.s*
Indoor					
PY LLIN	Baseline	0.148	0.081	− 0.030	− 0.032
	Post1	0.221	0.300	− 0.015	0.000
	Post2	0.220	0.218	− 0.048	− 0.051
	Baseline	0.136	− 0.078	− 0.031	− 0.039
PY-PPF LLIN	Post1	0.291	0.288	− 0.014	− 0.009
	Post2	0.055	− 0.029	− 0.067	− 0.068
PY-CFP LLIN	Baseline	0.166	0.169	− 0.037	− 0.030
	Post1	0.289	0.264	0.000	0.000
	Post2	0.269	0.388	− 0.039	− 0.065
Outdoor					
PY LLIN	Baseline	0.157	0.091	− 0.029	− 0.029
	Post1	0.207	0.322	0.000	− 0.035
	Post2	0.065	− 0.014	− 0.055	− 0.073
PY-PPF LLIN	Baseline	0.229	0.107	− 0.043	− 0.020
	Post1	0.458	0.564	− 0.016	− 0.008
	Post2	0.074	0.000	− 0.043	− 0.083
PY-CFP LLIN	Baseline	0.204	0.057	− 0.042	− 0.023
	Post1	0.291	0.264	− 0.028	− 0.016
	Post2	0.227	− 0.053	− 0.040	− 0.084

*An.* = *Anopheles gambiae* s.l.; PY LLIN: standard LLIN, LLIN treated with pyrethroid only; PY-CFP LLIN: LLIN bi-treated with pyrethroid-chlorfenapyr; PY-PPF LLIN: LLIN bi-treated with pyrethroid-pyriproxyfen; Post1 = 1st year post-intervention; Post2 = 2nd year post-intervention

In all *An. coluzzii* populations possessing the L1014F *vgsc-kdr* resistance allele, the *F*_*IS*_ values obtained were positive (*F*_*IS*_ > 0), showing a heterozygosity deficit that was observed before and after distribution of the LLINs to different study arms. Similar results were observed in *An. gambiae s.s.* populations except in the PY LLIN and PY-CFP LLIN arms post-intervention, where *F*_*IS*_ values were negative (*F*_*IS*_ < 0) signalling an excess of heterozygosity in these subpopulations. However, *Ho* was lower within *An. coluzzii* subpopulations compared with *He* indoors and outdoors in the different study arms before LLIN distribution (Table S1). In the two years after the intervention, a comparable trend was observed. In *An. gambiae s.s.*, before LLIN distribution, similar trends were obtained where *Ho* was also lower than *He*. After the intervention, *Ho* was slightly higher than *He* (Supplementary file, Table S5).

However, in all *An. coluzzii* populations possessing the G119S *ace-1* resistance allele, the *F*_*IS*_ values obtained were negative (*F*_*IS*_ < 0), showing an excess of heterozygosity observed before and after the study LLIN distributions. Similar results were observed in *An. gambiae s.s.* populations. However, the *F*_*IS*_ = 0 obtained mainly in the subpopulation of the PY-CFP LLIN arm suggests a lack of difference between observed and expected heterozygosity and was consistent with HWE (Table 5). *Ho* and *He* values before and after intervention in *An. coluzzii* subpopulations are very close, indicating agreement with panmictic expectations. Similar trends were obtained in *An. gambiae s.s.* subpopulations (Supplementary file, Table S6).

### Genetic differentiation in species of the *An. gambiae* complex

Differentiating between individuals before and after the distribution of LLINs, we observed a generally low genetic differentiation in indoor populations of *An. gambiae s.s.* with L1014F *vgsc-kdr* mutations, especially in the PY-PPF LLIN and PY-CFP LLIN arms (*F*_*ST*_ ≤ 0.05). By comparison, outside, genetic differentiation (*F*_*ST*_) was variable except in the PY-PPF LLIN arm where it was low (Table 4).

**Table 4:** Genetic differentiation in species of the *An. gambiae* complex

Location/LLINs	Period	Locus *vgsc*		Locus *Ace-1*	
		*F*_*ST*_		*F*_*ST*_	
		*An. coluzzii*	*An. gambiae s.s*	*An. coluzzii*	*An. gambiae s.s*
Indoor					
PY LLIN	Baseline	–	–	–	–
	Post1	0.9268d	< 0.001a	0.3517d	0.0109a
	Post2	0.0469a	0.2643d	0.3176d	0.3406d
PY-PPF LLIN	Baseline	–	–	–	–
	Post1	0.0084a	0.0066a	0.2425c	0.2611d
	Post2	0.1368b	0.0818b	0.0508a	0.2022c
PY-CFP LLIN	Baseline	–	–	–	–
	Post1	0.0273a	0.0019a	0.0028a	0.0275a
	Post2	< 0.0001a	0.0006a	1d	0.0763b
Outdoor					
PY LLIN	Baseline	–	–	–	–
	Post1	0.2706d	0.6165d	0.0861b	0.7595d
	Post2	0.0281a	< 0.0001a	0.2648d	0.1348b
PY-PPF LLIN	Baseline	–	–	–	–
	Post1	0.2367c	0.0108a	0.1918c	0.6974d
	Post2	0.3024d	< 0.0001a	1d	0.0501a
PY-CFP LLIN	Baseline	–	–	–	-
	Post1	0.0868b	0.1541c	0.6238d	1d
	Post2	0.0039a	0.0119b	1d	0.0297a

Letters a, b, c and d are distinct. a = low *F*_*ST*_, b = moderate *F*_*ST*_, c = high *F*_*ST*_ and d = very high *F*_*ST*_ within populations. *An.* = *Anopheles gambiae* s.l.; PY LLIN: standard LLIN, LLIN treated with pyrethroid only; PY-CFP LLIN: LLIN bi-treated with pyrethroid-chlorfenapyr; PY-PPF LLIN: LLIN bi-treated with pyrethroid-pyriproxyfen; Post1 = 1st year post-intervention; Post2 = 2nd year post-intervention

With G119S*-ace-1*, genetic differentiation was predominantly high (either *F*_*ST*_ = [0.15–0.25], or *F*_*ST*_ > 0.25) in both species (*An. gambiae s.s.* and *An. coluzzii*) except for subpopulations in the PY-CFP LLIN arm where they were low at year one post-intervention indoors. Similarly, in *An. gambiae s.s.,* a low genetic differentiation was observed during the second year in the PY-PPF and PY-CFP LLIN arms (Table 4).

### *Plasmodium falciparum* infection of vectors harbouring the L1014F-*vgsc-kdr* mutation

Overall, out of 2780 mosquitoes whose head-thoraxes were tested for *P. falciparum* infection, 93 mosquitoes were found to be positive. In *An. gambiae s.l*., the infection rate (IR) was 3.7% (95%CI: 2.8–4.7, 61/1659) indoors and 2.9% (95%CI: 1.9–3.9, 32/1138) outdoors (p = 0.25). Indoors, the IR was 4.8% (95%CI: 3.5–6.7, 36/743) in *An. gambiae s.s*. against 2.7% (95%CI: 1.8–4.1, 25/916) in *An. coluzzii* (p = 0.03). Outdoors, the IR was 3.9% (95%CI: 2.4–6.2, 18/464) in *An. gambiae s.s*. versus 2.1% (95%CI: 1.2–3.5, 14/674) in *An. coluzzii* (p = 0.1).

In *An. coluzzii*, there was no association between *P. falciparum* infection and genotype (p > 0.05 for all comparisons) in all study arms, with the exception of the 1st year following the intervention when “SS’’ genotypes were significantly more likely to be infected (Table 5). Similarly, no association (p > 0.05 for all comparisons) was observed in *An. gambiae s.s.* collected either indoors and outdoors (Table 5).

**Table 5:** Genotypic infection of *L1014F-vgsc*-*kdr* by *P. falciparum* in *Anopheles* species

Location/ species study arms	Periods	n positive	Genotypic infected (%)			χ^2^ Test	P-value
		(N total)	RR (n/N)	RS (n/N)	SS (n/N)		
Indoor (*An. coluzzii*)							
PY-PPF LLIN	Baseline	11 (218)	3.21 (7/158)	7.84 (4/51)	0 (0/9)	1.44	0.4877
	Post1	1 (148)	0 (0/92)	0 (0/37)	5.26 (1/19)	6.83	0.0327
	Post2	5 (120)	3.95 (3/76)	5.26 (2/38)	0 (0/6)	0.38	0.8251
PY-CFP LLIN	Baseline	5 (168)	3.84 (5/130)	0 (0/32)	0 (0/6)	1.51	0.4709
	Post1	1 (140)	0 (0/96)	0 (0/32)	8.33 (1/12)	10.74	0.0046
	Post2	2 (122)	1.35 (1/74)	2.94 (1/34)	0 (0/14)	0.62	0.7302
Outdoor (*An. coluzzii*)							
PY-PPF LLIN	Baseline	4 (123)	3.45 (3/87)	3.57 (1/28)	0 (0/4)	0.14	0.9301
	Post1	1 (99)	1.49 (1/67)	0 (0/21)	0 (0/11)	0.48	0.7857
	Post2	4 (117)	2.77 (2/72)	5.4 (2/37)	0 (0/8)	0.81	0.6653
PY-CFP LLIN	Baseline	2 (126)	2.22 (2/91)	0 (0/28)	0 (0/7)	0.78	0.6765
	Post1	1 (93)	1.69 (1/59)	0 (0/24)	0 (0/10)	0.58	0.7473
	Post2	2 (116)	3.03 (2/66)	0 (0/36)	0 (0/14)	1.54	0.4626
Indoor (*An. gambiae* s.s.)							
PY-PPF LLIN	Baseline	10 (174)	6.08 (9/148)	3.84 (1/26)	0 (0/0)	0	0
	Post1	5 (60)	6.82 (3/44)	16.67 (2/12)	0 (0/4)	1.58	0.4523
	Post2	2 (88)	2.43 (2/82)	0 (0/6)	0 (0/0)	0	0
PY-CFP LLIN	Baseline	13 (221)	6.11 (11/180)	5.71 (2/35)	0 (0/6)	0.39	0.8213
	Post1	2 (109)	2.7 (2/74)	0 (0/26)	0 (0/9)	0.96	0.6177
	Post2	4 (91)	4.59 (4/87)	0 (0/3)	0 (0/1)	0.19	0.9083
Outdoor (*An. gambiae* s.s.)							
PY-PPF LLIN	Baseline	4 (78)	6.56 (4/61)	0 (0/15)	0 (0/2)	1.17	0.5557
	Post1	2 (61)	4.87 (2/41)	0 (0/10)	0 (0/10)	1.01	0.6039
	Post2	3 (60)	5.08 (3/59)	0 (0/1)	0 (0/0)	0	0
PY-CFP LLIN	Baseline	3 (89)	4.41 (3/68)	0 (0/19)	0 (0/2)	0.95	0.6192
	Post1	1 (65)	2.22 (1/45)	0 (0/15)	0 (0/5)	0.45	0.798
	Post2	5 (94)	5.95 (5/84)	0 (0/10)	0 (0/0)	0	0

An*.: Anopheles*; N: number tested; n positive: number infected with *P. falciparum*, PY-CFP LLIN: LLIN bi-treated with pyrethroid-chlorfenapyr; PY-PPF LLIN: LLIN bi-treated with pyrethroid-pyriproxyfen; RR: homozygous resistant; RS: heterozygous resistant; SS: homozygous susceptible; [R]: frequency of resistant allele; [S]: frequency of susceptible allele; χ^2^ square: Chi-square test; Post1: 1st year post-intervention; Post2: 2nd year post-intervention

## Discussion

The present study provides information on the genetic diversity of *An. gambiae s.l.* populations in the communes of Covè-Zagnanado-Ouinhi where two types of bi-treated LLINs were distributed.

Of note, the present study was performed as part of a large randomized controlled trial during which, over the two first years, both PY-CFP LLIN and PY-PPF LLIN reduced significantly the indoor entomological inoculation rate (EIR) by 66% (p = 0.0005) and 58% (p = 0.0028) respectively, while only PY-CFP LLIN significantly reduced the outdoor EIR by 70% (0.0035) [38]. Moreover, both PY-CFP LLIN and PY-PPF LLIN were found to perform similarly on the density of the two primary vectors (*An. gambiae s.s.* and *An. coluzzii*) as compared to PY LLIN [39]. In all the three study arms, there was a significant decrease in pyrethroid resistance intensity in *An. gambiae s.l*. in the first-year compared to the baseline, while a significant increase was observed in the second year compared to the first one [40].

Overall, findings of the present study revealed that the frequency of the L1014F *vgsc-kdr* resistance allele in the two molecular species remained high (over 70%) in the three study arms, pre- and post-intervention. Also, similar *kdr* frequencies were observed between the PY-PPF LLIN and PY-CFP- LLIN arms and the PY LLIN arm (control arm) in both molecular species and over years (Supplementary file, Table S2). A decrease in this frequency after the intervention was observed, which is not a common phenomenon as the opposite trend has occurred in several settings and was attributed to either the pressure of insecticide contamination in the soil after agricultural practices [41], or the use of ITNs [42–44]. This trend suggests the possibility of interactions between pyrethroids and the new active ingredients (chlorfenapyr and pyriproxyfen), though cross-resistance seems unlikely.

The frequency of the G119S*-ace-1* allele was low in both species. This is not unexpected, as this mutation is associated with resistance to carbamates and organophosphates [34, 45], which have not been deployed in the study area. Moreover, none of the new chemicals (chlorfenapyr and pyriproxyfen) are cross-resistant to carbamates and organophosphates at least via the G119S*-Ace-1* mutation. This mutation often comes with fitness cost, requiring intense selection pressure prior to high occurrence [46]. Several other studies documented the low frequency of this mutation in different settings in Benin [6, 7, 47, 48].

A deficit of heterozygosity (*F*_*IS*_ > 0) was observed in populations of *An. coluzzii* possessing the L1014F*-vgsc-kdr* mutation. This observation may reflect the effect of pyrethroid insecticides from study LLINs, or other unknown insecticides deployed on mosquitoes, which would continue to maintain resistance within populations by increasing the number of homozygous resistant individuals. This phenomenon could also lead to the elimination of susceptible individuals in various populations, favouring the survival of resistant ones. During the second year, an excess of heterozygosity was observed within the *An. gambiae s.s.* populations in the three study arms, which contrasts with findings from the recent work of Fassinou et al*.* [49]. This may simply be a random variation from one year to the next. Variations in population size, demographic composition, migration and population seasonality [50] can all influence levels of heterozygosity.

Low genetic differentiation was observed indoors post-intervention in the PY-PPF LLIN and PY-CFP LLIN arms in the two species carrying the L1014F*-vgsc-kdr* mutation. This could account for the continued increased genetic homogeneity in resistant *An. gambiae s.l.* populations following the widespread use of these new study LLINs. In a cotton-growing area in Benin, Aïkpon et al*.* [51] showed little genetic differentiation between *An. gambiae s.s*. populations at the L1014F *vgsc-kdr* and G119S*-ace-1* loci. Selective pressures exerted by insecticides may favour the survival of individuals carrying resistance alleles. However, in the present study, genetic differentiation within the two species with the G119S*-ace-1* mutation remained generally high after the distribution of the LLINs in the study. Thus, this results could rule out any selection of the G119S*-ace-1* mutation; since no IRS has occurred in the study area to date. Ngufor et al. [52] evaluated in a community trial Fludora^®^ Fusion, (mixture of deltamethrin and clothianidin) and VECTRON™ T500 (TENEBENAL™) in the neigbouring commune of Za-kpota, observing that G119S*-ace-1* allele frequencies decreased from 36 to 10% in the VECTRON™ T500 arm and from 15 to 11% in the Fludora^®^ Fusion arm. Moreover, some studies have reported an increase in L1014F vgsc-*kdr* mutation frequencies in *An. gambiae s.s.* and *An. coluzzii* species in response to net distribution in some areas [27, 40, 53]. These results provide some evidence of the effect of the introduction of new insecticides on frequencies of resistance mutations. In the large RCT, the infection rate in *An. gambiae s.l.* was 1% in both PY-CFP LLIN and PY-PPF LLIN arms over the two first years [38]. Moreover, it has been shown in wild mosquitoes possessing the resistance allele at the L1014F *vgsc-kdr* locus, that the risks of being infected by oocysts and sporozoites were higher in the *“RR”* and “*RS”* genotypes compared to “*SS*” [54]. However, in the present study, there was no evidence of a difference in the infection rate among the three genotypes, which deserves further investigation.

The study period (two years after the intervention) is short for an in-depth study of the genetic behaviour of the species. Similarly, the restricted geographical area of the study, limited to just three districts and randomized by village, is a potential limitation for the study as the flight of mosquito vectors between habitats could result in significant gene flow, thereby reducing the genetic differentiation between populations. Further analysis, including additional genetic data obtained 4 to 5 years after the deployment of these new-generation LLINs at scale, could provide additional information on the impact of dual AI nets on phenotypic resistance to pyrethroids and their impact on the underlying population genetic structure of malaria vectors.

## Conclusion

The L1014F *vgsc-kdr* resistance allele showed a high allele frequency, while a low frequency was observed for the G119S *ace-1* allele. This high frequency of L1014F *vgsc-kdr* allele indicates that dual AI nets continue to exert selective pressure in favour of this allele that is not counteracted by the non-pyrethroid insecticide.

## Supplementary Information

Additional file 1: Table S1. Genotypic and allelic frequenciesof L1014F and HWE test indoor of *Anopheles gambiae* *s.s.* and *Anopheles coluzzii* populations. *An.: Anopheles*; N: number tested; PY LLIN: standard LLIN, LLIN treated with pyrethroid only; PY-CFP LLIN: LLIN bi-treated with pyrethroid-chlorfenapyr; PY-PPF LLIN: LLIN bi-treated with pyrethroid-pyriproxyfen; RR: homozygous resistant; RS: heterozygous resistant; SS: homozygous susceptible; Frfrequency of resistance allele; p valuep value to the Hardy–Weinberg Equilibrium; Post1: 1st year post-intervention; Post2: 2nd year post-intervention

Additional file 2: Table S2. Allele frequency of the L1014F *vgsc*-*kdr* mutation in *An. gambiae s.s.* and *An. coluzzii* in the three study arms. *An.: Anopheles*; N: number tested; PY LLIN: standard LLIN, LLIN treated with pyrethroid only; PY-CFP LLIN: LLIN bi-treated with pyrethroid-chlorfenapyr; PY-PPF LLIN: LLIN bi-treated with pyrethroid-pyriproxyfen; Post1: 1st year post-intervention; Post2; 2nd year post-intervention, CI: confidence interval

Additional file 3: Table S3. Genotypic and allelic frequenciesof G119S and HWE test outdoor of *Anopheles gambiae* *s.s.* and *Anopheles coluzzii* populations. *An.: Anopheles*; N: number tested; PY LLIN: standard LLIN, LLIN treated with pyrethroid only; PY-CFP LLIN: LLIN bi-treated with pyrethroid-chlorfenapyr; PY-PPF LLIN: LLIN bi-treated with pyrethroid-pyriproxyfen; RR: homozygous resistant; RS: heterozygous resistant; SS: homozygous susceptible; p value: p value for Hardy–Weinberg Equilibrium; Post1: 1st year post-intervention; Post2: 2nd year post-intervention

Additional file 4: Table S4. Allele frequency of the G119S-*ace-1* mutation in *An. gambiae s.s.* and *An. coluzzii* in the three study arms. *An.: Anopheles*; N: number tested; PY LLIN: standard LLIN, LLIN treated with pyrethroid only; PY-CFP LLIN: LLIN bi-treated with pyrethroid-chlorfenapyr; PY-PPF LLIN: LLIN bi-treated with pyrethroid-pyriproxyfen; Post1: 1st year post-intervention; Post2: 2nd year post-intervention, CI: confidence interval

Additional file 5: Table S5. Expected heterozygousand observed heterozygouswithin the locus L1014F*-vgsc-kdr* in *An. gambiae s.s.* and *An. coluzzii* species. *An.: Anopheles*; N: number tested; PY LLIN: standard LLIN, LLIN treated with pyrethroid only; PY-CFP LLIN: LLIN bi-treated with pyrethroid-chlorfenapyr; PY-PPF LLIN: LLIN bi-treated with pyrethroid-pyriproxyfen; Post1: 1st year post-intervention; Post2: 2nd year post-intervention.

Additional file 6: Table S6. Expected heterozygousand observed heterozygouswithin the locus *Ace*-*1* in *An. gambiae s.s* and *An. coluzzii* species. *An.: Anopheles gambiae* s.l.; N: number tested; PY LLIN: standard LLIN, LLIN treated with pyrethroid only; PY-CFP LLIN: LLIN bi-treated with pyrethroid-chlorfenapyr; PY-PPF LLIN: LLIN bi-treated with pyrethroid-pyriproxyfen; Post1: 1st year post-intervention; Post2: 2nd year post-intervention.

## Data Availability

No datasets were generated or analysed during the current study.

## Abbreviations

RCT: Randomized controlled trial
AI: Active-ingredients
*Vgsc*: Voltage-gated sodium channel
*kdr*: Knock down resistance
ace: Acetylcholinesterase
IRS: Indoor residual spraying
LLINs: Long-lasting insecticidal nets
HWE: Hardy–Weinberg equilibrium
Ho: Observed heterozygosity
He: Expected heterozygosity
*F*_*IS*_: Fixation index
PY-PPF: Pyrethroid-pyriproxyfen
PY-CFP: Pyrethroid-chlorfenapyr
ITNs: Insecticide-treated nets
PBO: Piperonyl butoxide
HLC: Human landing catch
ELISA: Enzyme-linked immuno sorbent assay
CSP: Circum-sporozoite protein

## References

[1] WHO. World malaria report 2020: 20 years of global progress and challenges. Geneva, World Health Organization. Available from: https://apps.who.int/iris/handle/10665/337660.

[2] Araújo MF, Castanheira EMS, Sousa SF. The buzz on insecticides: a review of uses, molecular structures, targets, adverse effects, and alternatives. Molecules. 2023;28:3641. 37110875 10.3390/molecules28083641 PMC10144373

[3] Costa LG. The neurotoxicity of organochlorine and pyrethroid pesticides. In: Lotti M, Bleecker ML (eds.). Handbook of Clinical Neurology. Elsevier; 2015 [cited 2024 Jun 22]. Chapt. 9; 135–48. Available from: https://www.sciencedirect.com/science/article/pii/B9780444626271000093 10.1016/B978-0-444-62627-1.00009-3 26563787

[4] Čolović MB, Krstić DZ, Lazarević-Pašti TD, Bondžić AM, Vasić VM. Acetylcholinesterase inhibitors: pharmacology and toxicology. Curr Neuropharmacol. 2013;11:315–35. 24179466 10.2174/1570159X11311030006 PMC3648782

[5] Yovogan B, Sovi A, Padonou GG, Adoha CJ, Akinro B, Chitou S, et al. Pre-intervention characteristics of the mosquito species in Benin in preparation for a randomized controlled trial assessing the efficacy of dual active-ingredient long-lasting insecticidal nets for controlling insecticide-resistant malaria vectors. PLoS ONE. 2021;16: e0251742. 34014982 10.1371/journal.pone.0251742 PMC8136630

[6] Salako AS, Ahogni I, Aïkpon R, Sidick A, Dagnon F, Sovi A, et al. Insecticide resistance status, frequency of L1014F Kdr and G119S Ace-1 mutations, and expression of detoxification enzymes in *Anopheles gambiae* (s.l.) in two regions of northern Benin in preparation for indoor residual spraying. Parasit Vectors. 2018;11:618. 30509288 10.1186/s13071-018-3180-2 PMC6278060

[7] Kpanou CD, Sagbohan HW, Dagnon F, Padonou GG, Ossè R, Salako AS, et al. Characterization of resistance profile (intensity and mechanisms) of *Anopheles gambiae* in three communes of northern Benin, West Africa. Malar J. 2021;20:328. 34315480 10.1186/s12936-021-03856-2 PMC8314583

[8] N’Guessan R, Corbel V, Akogbéto M, Rowland M. Reduced efficacy of insecticide-treated nets and indoor residual spraying for malaria control in pyrethroid resistance area, Benin. Emerg Infect Dis. 2007;13:199–206. 17479880 10.3201/eid1302.060631 PMC2725864

[9] Grau-Bové X, Lucas E, Pipini D, Rippon E, van’t Hof AE, Constant E, et al. Resistance to pirimiphos-methyl in West African *Anopheles* is spreading via duplication and introgression of the Ace1 locus. PLoS Genet. 2021;17:e1009253. 33476334 10.1371/journal.pgen.1009253 PMC7853456

[10] Kouamé RMA, Lynd A, Kouamé JKI, Vavassori L, Abo K, Donnelly MJ, et al. Widespread occurrence of copy number variants and fixation of pyrethroid target site resistance in *Anopheles gambiae* (s.l.) from southern Côte d’Ivoire. Curr Res Parasitol Vector Borne Dis. 2023;3:100117. 36970448 10.1016/j.crpvbd.2023.100117 PMC10031352

[11] Martinez-Torres D, Chandre F, Williamson MS, Darriet F, Bergé JB, Devonshire AL, et al. Molecular characterization of pyrethroid knockdown resistance (kdr) in the major malaria vector *Anopheles gambiae* s.s. Insect Mol Biol. 1998;7:179–84. 9535162 10.1046/j.1365-2583.1998.72062.x

[12] Sovi A, Djègbè I, Soumanou L, Tokponnon F, Gnanguenon V, Azondékon R, et al. Microdistribution of the resistance of malaria vectors to deltamethrin in the region of Plateau (southeastern Benin) in preparation for an assessment of the impact of resistance on the effectiveness of long lasting insecticidal nets (LLINs). BMC Infect Dis. 2014;14:103. 24564260 10.1186/1471-2334-14-103 PMC3941697

[13] Aïkpon R, Agossa F, Ossè R, Oussou O, Aïzoun N, Oké-Agbo F, et al. Bendiocarb resistance in *Anopheles gambiae* s.l. populations from Atacora department in Benin, West Africa: a threat for malaria vector control. Parasit Vectors. 2013;6:192. 23803527 10.1186/1756-3305-6-192 PMC3698110

[14] Djogbénou L, Pasteur N, Akogbéto M, Weill M, Chandre F. Insecticide resistance in the *Anopheles gambiae* complex in Benin: a nationwide survey. Med Vet Entomol. 2011;25:256–67. 21155858 10.1111/j.1365-2915.2010.00925.x

[15] Djègbè I, Boussari O, Sidick A, Martin T, Ranson H, Chandre F, et al. Dynamics of insecticide resistance in malaria vectors in Benin: first evidence of the presence of L1014S kdr mutation in *Anopheles gambiae* from West Africa. Malar J. 2011;10:261. 21910856 10.1186/1475-2875-10-261 PMC3179749

[16] WHO. World malaria report 2012 [Internet]. Geneva, World Health Organization, 2012 [cited 2024 Jan 24]. Available from: https://www.who.int/publications-detail-redirect/9789241564533

[17] Djouaka RF, Bakare AA, Coulibaly ON, Akogbeto MC, Ranson H, Hemingway J, et al. Expression of the cytochrome P450s, CYP6P3 and CYP6M2 are significantly elevated in multiple pyrethroid resistant populations of *Anopheles gambiae* s.s. from Southern Benin and Nigeria. BMC Genomics. 2008;9:538. 19014539 10.1186/1471-2164-9-538 PMC2588609

[18] Oumbouke WA, Rowland M, Koffi AA, Alou LPA, Camara S, N’Guessan R. Evaluation of an alpha-cypermethrin + PBO mixture long-lasting insecticidal net VEERALIN® LN against pyrethroid resistant *Anopheles gambiae* s.s.: an experimental hut trial in M’bé, central Côte d’Ivoire. Parasit Vectors. 2019;12:544. 31730481 10.1186/s13071-019-3796-x PMC6858630

[19] Toe KH, Müller P, Badolo A, Traore A, Sagnon N, Dabiré RK, et al. Do bednets including piperonyl butoxide offer additional protection against populations of *Anopheles gambiae* s.l. that are highly resistant to pyrethroids? An experimental hut evaluation in Burkina Fasov. Med Vet Entomol. 2018;32:407–16. 29998497 10.1111/mve.12316

[20] Sagbohan HW, Kpanou CD, Sovi A, Osse R, Sidick A, Adoha C, et al. Pyrethroid resistance intensity in *Anopheles gambiae* s.l. from different agricultural production zones in Benin, West Africa. Vector Borne Zoonotic Dis. 2022;22:39–47. 35030048 10.1089/vbz.2021.0066

[21] WHO. World malaria report 2023 [Internet]. Geneva, World Health Organization, 2023 [cited 2024 Jan 24]. Available from: https://www.who.int/teams/global-malaria-programme/reports/world-malaria-report-2023

[22] Bayili K, Ndo S, Namountougou M, Sanou R, Ouattara A, Dabiré RK, et al. Evaluation of efficacy of Interceptor® G2, a long-lasting insecticide net coated with a mixture of chlorfenapyr and alpha-cypermethrin, against pyrethroid resistant *Anopheles gambiae* s.l. in Burkina Faso. Malar J. 2017;16:190. 28482891 10.1186/s12936-017-1846-4 PMC5422893

[23] Camara S, Alou LPA, Koffi AA, Clegban YCM, Kabran J-P, Koffi FM, et al. Efficacy of Interceptor® G2, a new long-lasting insecticidal net against wild pyrethroid-resistant *Anopheles gambiae* s.s. from Côte d’Ivoire: a semi-field trial. Parasite. 2018;25:42. 30088473 10.1051/parasite/2018042 PMC6082037

[24] Black BC, Hollingworth RM, Ahammadsahib KI, Kukel CD, Donovan S. Insecticidal action and mitochondrial uncoupling activity of AC-303,630 and related halogenated pyrroles. Pesticide Biochem Physiol. 1994;50:115–28.

[25] Ishaaya I, Horowitz AR. Insecticides with novel modes of action: an overview. In: Ishaaya I, Degheele D, editors. Insecticides with novel modes of action: mechanisms and application. Berlin: Springer-Verlag; 1998.

[26] Sánchez-Ramos I, Fernández CE, González-Núñez M. Laboratory evaluation of insect growth regulators against the spotted wing drosophila. Drosophila suzukii J Pest Sci. 2023;97:885–95. 10.1093/jee/toaf285 41234041

[27] Norris LC, Main BJ, Lee Y, Collier TC, Fofana A, Cornel AJ, et al. Adaptive introgression in an African malaria mosquito coincident with the increased usage of insecticide-treated bed nets. Proc Natl Acad Sci USA. 2015;112:815–20. 25561525 10.1073/pnas.1418892112 PMC4311837

[28] Odjo EM, Salako AS, Padonou GG, Yovogan B, Adoha CJ, Adjottin B, et al. What can be learned from the residual efficacy of three formulations of insecticides (pirimiphos-methyl, clothianidin and deltamethrin mixture, and clothianidin alone) in large-scale in community trial in North Benin, West Africa? Malar J. 2023;22:150. 37158866 10.1186/s12936-023-04572-9 PMC10165746

[29] Accrombessi M, Cook J, Ngufor C, Sovi A, Dangbenon E, Yovogan B, et al. Assessing the efficacy of two dual-active ingredients long-lasting insecticidal nets for the control of malaria transmitted by pyrethroid-resistant vectors in Benin: study protocol for a three-arm, single-blinded, parallel, cluster-randomized controlled trial. BMC Infect Dis. 2021;21:194. 33607958 10.1186/s12879-021-05879-1 PMC7892705

[30] Accrombessi M, Akogbeto MC, Dangbenon E, Akpovi H, Sovi A, Yovogan B, et al. Malaria burden and associated risk factors in an area of pyrethroid-resistant vectors in Southern Benin. Am J Trop Med Hyg. 2022;107:681–8. 35895353 10.4269/ajtmh.22-0190 PMC9490648

[31] Coetzee M. Key to the females of Afrotropical *Anopheles* mosquitoes (Diptera: Culicidae). Malar J. 2020;19:70. 32054502 10.1186/s12936-020-3144-9 PMC7020601

[32] Wirtz RA, Zavala F, Charoenvit Y, Campbell GH, Burkot TR, Schneider I, et al. Comparative testing of monoclonal antibodies against *Plasmodium falciparum* sporozoites for ELISA development. Bull World Health Organ. 1987;65:39–45. 3555879 PMC2490858

[33] Santolamazza F, Mancini E, Simard F, Qi Y, Tu Z, della Torre A. Insertion polymorphisms of SINE200 retrotransposons within speciation islands of *Anopheles gambiae* molecular forms. Malar J. 2008;7:163. 18724871 10.1186/1475-2875-7-163 PMC2546427

[34] Weill M, Malcolm C, Chandre F, Mogensen K, Berthomieu A, Marquine M, et al. The unique mutation in ace-1 giving high insecticide resistance is easily detectable in mosquito vectors. Insect Mol Biol. 2004;13:1–7. 14728661 10.1111/j.1365-2583.2004.00452.x

[35] Weir BS, Cockerham CC. Estimating F-statistics for the analysis of population structure. Evolution. 1984;38:1358–70. 28563791 10.1111/j.1558-5646.1984.tb05657.x

[36] Robertson A, Hill WG. Deviations from Hardy-Weinberg proportions: sampling variances and use in estimation of inbreeding coefficients. Genetics. 1984;107:703–18. 6745643 10.1093/genetics/107.4.703 PMC1202385

[37] Hartl FU, Hlodan R, Langer T. Molecular chaperones in protein folding: the art of avoiding sticky situations. Trends Biochem Sci. 1994;19:20–5. 7908149 10.1016/0968-0004(94)90169-4

[38] Accrombessi M, Cook J, Dangbenon E, Yovogan B, Akpovi H, Sovi A, et al. Efficacy of pyriproxyfen-pyrethroid long-lasting insecticidal nets (LLINs) and chlorfenapyr-pyrethroid LLINs compared with pyrethroid-only LLINs for malaria control in Benin: a cluster-randomised, superiority trial. Lancet. 2023;401:435–46. 36706778 10.1016/S0140-6736(22)02319-4

[39] Yovogan B, Sovi A, Djènontin A, Adoha CJ, Akinro B, Accrombessi M, et al. The impact of pyrethroid-pyriproxyfen and pyrethroid-chlorfenapyr long-lasting insecticidal nets on density of primary malaria vectors *Anopheles gambiae* s.s. and *Anopheles coluzzii* in Benin: a secondary analysis of a cluster randomised controlled trial. Parasit Vectors. 2024;17:7. 38178161 10.1186/s13071-023-06104-5 PMC10768265

[40] Sovi A, Adoha CJ, Yovogan B, Cross CL, Dee DP, Konkon AK, et al. The effect of next-generation, dual-active-ingredient, long-lasting insecticidal net deployment on insecticide resistance in malaria vectors in Benin: results of a 3-year, three-arm, cluster-randomised, controlled trial. Lancet Planet Health. 2024;8:e894-905. 39515347 10.1016/S2542-5196(24)00232-8

[41] Luc DS, Benoit A, Laurette D, Michel M. Indirect evidence that agricultural pesticides select for insecticide resistance in the malaria vector *Anopheles gambiae*. J Vector Ecol. 2016;41:34–40. 27232122 10.1111/jvec.12191

[42] Assogba BS, Pasteur N, Makoundou P, Unal S, Baba-Moussa L, Labbé P, et al. Dynamic of resistance alleles of two major insecticide targets in *Anopheles gambiae* (s.l.) populations from Benin, West Africa. Parasit Vectors. 2020;13:134. 32171326 10.1186/s13071-020-4006-6 PMC7071764

[43] Metelo-Matubi E, Zanga J, Binene G, Mvuama N, Ngamukie S, Nkey J, et al. The effect of a mass distribution of insecticide-treated nets on insecticide resistance and entomological inoculation rates of *Anopheles gambiae* s.l. in Bandundu City, Democratic Republic of Congo. Pan Afr Med J. 2021;40:118. 34887992 10.11604/pamj.2021.40.118.27365 PMC8627145

[44] Sanou A, Nelli L, Guelbéogo WM, Cissé F, Tapsoba M, Ouédraogo P, et al. Insecticide resistance and behavioural adaptation as a response to long-lasting insecticidal net deployment in malaria vectors in the Cascades region of Burkina Faso. Sci Rep. 2021;11:17569. 34475470 10.1038/s41598-021-96759-w PMC8413378

[45] Essandoh J, Yawson AE, Weetman D. Acetylcholinesterase (Ace-1) target site mutation 119S is strongly diagnostic of carbamate and organophosphate resistance in *Anopheles gambiae* s.s. and Anopheles coluzzii across southern Ghana. Malar J. 2013;12:404. 24206629 10.1186/1475-2875-12-404 PMC3842805

[46] Assogba BS, Djogbénou LS, Milesi P, Berthomieu A, Perez J, Ayala D, et al. An ace-1 gene duplication resorbs the fitness cost associated with resistance in *Anopheles gambiae*, the main malaria mosquito. Sci Rep. 2015;5:14529. 26434951 10.1038/srep14529 PMC4592963

[47] Yadouleton AW, Padonou G, Asidi A, Moiroux N, Bio-Banganna S, Corbel V, et al. Insecticide resistance status in *Anopheles gambiae* in southern Benin. Malar J. 2010;9:83. 20334637 10.1186/1475-2875-9-83 PMC2858214

[48] Kpanou CD, Sagbohan HW, Sovi A, Osse R, Padonou GG, Salako A, et al. Assessing insecticide susceptibility and resistance intensity of *Anopheles gambiae* s.l. populations from some districts of Benin Republic, West Africa. J Med Entomol. 2022;59:949–56. 35357491 10.1093/jme/tjac037

[49] Fassinou AJYH, Koukpo CZ, Ossè RA, Agossa FR, Assogba BS, Sidick A, et al. Genetic structure of *Anopheles gambiae* s.s populations following the use of insecticides on several consecutive years in southern Benin. Trop Med Health. 2019;47:23. 31007534 10.1186/s41182-019-0151-z PMC6458727

[50] Johnson OL, Tobler R, Schmidt JM, Huber CD. Fluctuating selection and the determinants of genetic variation. Trends Genet. 2023;39:491–504. 36890036 10.1016/j.tig.2023.02.004

[51] Aikpon R, Missihoun A, Lokossou A, Aikpon G, Salifou S, Dansi A, et al. Hétérogénéité génétique et résistance des vecteurs du paludisme (*Anopheles gambiae* s.l) aux insecticides en zone cotonnière au Benin. Int J Biol Chem Sci. 2020;14:2724–36.

[52] Ngufor C, Govoetchan R, Fongnikin A, Hueha C, Ahoga J, Syme T, et al. Community evaluation of VECTRON™ T500, a broflanilide insecticide, for indoor residual spraying for malaria vector control in central Benin; a two arm non-inferiority cluster randomised trial. Sci Rep. 2023;13:17852. 37857762 10.1038/s41598-023-45047-w PMC10587144

[53] Mandeng SE, Awono-Ambene HP, Bigoga JD, Ekoko WE, Binyang J, Piameu M, et al. Spatial and temporal development of deltamethrin resistance in malaria vectors of the *Anopheles gambiae* complex from North Cameroon. PLoS ONE. 2019;14: e0212024. 30779799 10.1371/journal.pone.0212024 PMC6380565

[54] Ndiath MO, Cailleau A, Diedhiou SM, Gaye A, Boudin C, Richard V, et al. Effects of the *kdr* resistance mutation on the susceptibility of wild *Anopheles gambiae* populations to *Plasmodium falciparum:* a hindrance for vector control. Malar J. 2014;13:340. 25176292 10.1186/1475-2875-13-340 PMC4159551

