# Supplementary material for: Impact of dual active ingredients long-lasting insecticidal nets on the genetic structure of insecticide resistant populations of Anopheles gambiae in Southern Benin
Source: Malar J. 2025 Mar 4;24:72. doi: 10.1186/s12936-025-05308-7 (PMC11877869; doi:10.1186/s12936-025-05308-7)
Supplement: Supplementary file 1 — Additional file 1: Table S1. Genotypic and allelic frequenciesof L1014F and HWE test indoor of Anopheles gambiae s.s. and Anopheles coluzzii populations. An.: Anopheles; N: number tested; PY LLIN: standard LLIN, LLIN treated with pyrethroid only; PY-CFP LLIN: LLIN bi-treated with pyrethroid-chlorfenapyr; PY-PPF LLIN: LLIN bi-treated with pyrethroid-pyriproxyfen; RR: homozygous resistant; RS: heterozygous resistant; SS: homozygous susceptible; Frfrequency of resistance allele; p valuep value to the Hardy–Weinberg Equilibrium; Post1: 1st year post-intervention; Post2: 2nd year post-intervention [file 12936_2025_5308_MOESM1_ESM.docx]

**Table S1 :** Genotypic and allelic frequencies (Fr) of L1014F and HWE test Outdoor of *Anopheles gambiae* s.s. and *Anopheles coluzzii* populations

|  |  |  |  | **Outdoor** |  |  |  |
| --- | --- | --- | --- | --- | --- | --- | --- |
| **Period/ species** | **Study arms** | **N *An*.** | **Genotypic frequencies** | | | **Fr (L1014F)** | **P- value (HWE)** |
|  |  |  | **RR (%)** | **RS (%)** | **SS (%)** |  |  |
| **Baseline** |  |  |  |  |  |  |  |
| *An. coluzzii* | PY LLIN | 125 | 98 (78.4) | 23 (18.4) | 4 (4.1) | 87,6 | 0.0946 |
|  | PY-PPF LLIN | 123 | 87 (70.7) | 28 (22.8) | 8 (9.2) | 82,1 | 0.0145 |
|  | PY-CFP LLIN | 126 | 91 (72.2) | 28 (22.2) | 7 (7.7) | 83,3 | 0.0298 |
| *An. gambiae* s.s. | PY LLIN | 90 | 66 (73.3) | 21 (23.3) | 3 (4.6) | 85,0 | 0.3080 |
|  | PY-PPF LLIN | 78 | 61 (78.2) | 15 (19.2) | 2 (3.3) | 87,8 | 0.3000 |
|  | PY-CFP LLIN | 89 | 68 (76.4) | 19 (21.4) | 2 (2.9) | 87,1 | 0.4346 |
| **Post1** | | |  |  |  |  |  |
| *An. coluzzii* | PY LLIN | 91 | 66 (72.5) | 20 (22.0) | 5 (7.6) | 83,5 | 0.0633 |
|  | PY-PPF LLIN | 99 | 67 (67.7) | 21 (21.2) | 11 (16.4) | 78,3 | p<0.001 |
|  | PY-CFP LLIN | 93 | 59 (63.4) | 24 (25.8) | 10 (16.95) | 76,3 | 0.0078 |
| *An. gambiae* s.s. | PY LLIN | 60 | 48 (80) | 9 (15.0) | 3 (6.3) | 87,5 | 0.0359 |
|  | PY-PPF LLIN | 61 | 41 (67.2) | 10 (16.4) | 10 (24.4) | 75,4 | p<0.001 |
|  | PY-CFP LLIN | 65 | 45 (69.2) | 15 (23.1) | 5 (11.1) | 80,8 | 0.0450 |
| **Post2** | | |  |  |  |  |  |
| *An. coluzzii* | PY LLIN | 99 | 64 (64.7) | 30 (30.3) | 5 (7.8) | 79,8 | 0.3506 |
|  | PY-PPF LLIN | 117 | 72 (61.5) | 37 (31.6) | 8 (11.1) | 77,4 | 0.2912 |
|  | PY-CFP LLIN | 116 | 66 (56.9) | 36 (31.0) | 14 (21.2) | 72,4 | 0.0152 |
| *An. gambiae* s.s. | PY LLIN | 75 | 72 (96.0) | 3 (4.0) | 0 (0) | 98,0 | 1 |
|  | PY-PPF LLIN | 60 | 59 (98.3) | 1 (1.7) | 0 (0) | 99,2 | - |
|  | PY-CFP LLIN | 111 | 99 (89.2) | 12 (10.8) | 0 (0) | 94,6 | 1 |

*An.: Anopheles*; N: number tested; PY LLIN: standard LLIN, LLIN treated with pyrethroid only; PY-CFP LLIN: LLIN bi-treated with pyrethroid-chlorfenapyr; PY-PPF LLIN: LLIN bi-treated with pyrethroid-pyriproxyfen; RR: homozygous resistant; RS: heterozygous resistant; SS: homozygous susceptible; Fr (R) frequency of resistance allele; p value (HWE): p value to the Hardy-Weinberg Equilibrium; Post1: 1st year post-intervention; Post2: 2nd year post-intervention
