# Supplementary material for: Impact of dual active ingredients long-lasting insecticidal nets on the genetic structure of insecticide resistant populations of Anopheles gambiae in Southern Benin
Source: Malar J. 2025 Mar 4;24:72. doi: 10.1186/s12936-025-05308-7 (PMC11877869; doi:10.1186/s12936-025-05308-7)
Supplement: Supplementary file 2 — Additional file 2: Table S2. Allele frequency of the L1014F vgsc-kdr mutation in An. gambiae s.s. and An. coluzzii in the three study arms. An.: Anopheles; N: number tested; PY LLIN: standard LLIN, LLIN treated with pyrethroid only; PY-CFP LLIN: LLIN bi-treated with pyrethroid-chlorfenapyr; PY-PPF LLIN: LLIN bi-treated with pyrethroid-pyriproxyfen; Post1: 1st year post-intervention; Post2; 2nd year post-intervention, CI: confidence interval [file 12936_2025_5308_MOESM2_ESM.docx]

**Table S2 : Allele frequency of the L1014F *vgsc*-*Kdr* mutation in *An. gambiae* s.s. and *An. coluzzii* in the three study arms**

|  |  | **Indoor** | | | | | |  | **Outdoor** | | | | | |
| --- | --- | --- | --- | --- | --- | --- | --- | --- | --- | --- | --- | --- | --- | --- |
| **Period/Molecular species** | **Study arms** | **N *An.*** | **RR** | **RS** | **SS** | **Fr (L1014F)** | **95% CI** |  | **N *An.*** | **RR** | **RS** | **SS** | **Fr (L1014F)** | **95% CI** |
| **Baseline** |  |  |  |  |  |  |  |  |  |  |  |  |  |  |
| *An. coluzzii* | PY LLIN | 208 | 145 | 52 | 11 | 82.2 | 78.1-85.7 |  | 125 | 98 | 23 | 4 | 87.9 | 82.7-91.3 |
|  | PY-PPF LLIN | 218 | 158 | 51 | 9 | 84.2 | 80.3-87.4 |  | 123 | 87 | 28 | 8 | 82.1 | 76.6-86.6 |
|  | PY-CFP LLIN | 168 | 130 | 32 | 6 | 86.9 | 82.7-90.2 |  | 126 | 91 | 28 | 7 | 83.3 | 78-87.6 |
| *An. gambiae* s.s. | PY LLIN | 177 | 152 | 23 | 2 | 92.4 | 88.9-94.8 |  | 90 | 66 | 21 | 3 | 85 | 78.7-89.7 |
|  | PY-PPF LLIN | 174 | 148 | 26 | 0 | 92.5 | 89.1-94.9 |  | 78 | 61 | 15 | 2 | 87.8 | 81.4-92.3 |
|  | PY-CFP LLIN | 221 | 180 | 35 | 6 | 89.4 | 86.0-92.0 |  | 89 | 68 | 19 | 2 | 87.1 | 81-91.5 |
| **Post1** | | |  |  |  |  |  |  |  |  |  |  |  |  |
| *An. coluzzii* | PY LLIN | 167 | 117 | 39 | 11 | 81.7 | 77.1-85.6 |  | 91 | 66 | 20 | 5 | 83.5 | 77.1-88.4 |
|  | PY-PPF LLIN | 148 | 92 | 37 | 19 | 74.7 | 69.2-79.4 |  | 99 | 67 | 21 | 11 | 78.3 | 71.7-83.7 |
|  | PY-CFP LLIN | 140 | 96 | 32 | 12 | 80 | 74.7-84.4 |  | 93 | 59 | 24 | 10 | 76.3 | 69.4-82.1 |
| *An. gambiae* s.s. | PY LLIN | 124 | 78 | 32 | 14 | 75.8 | 69.9-80.9 |  | 60 | 48 | 9 | 3 | 87.5 | 79.9-92.6 |
|  | PY-PPF LLIN | 60 | 44 | 12 | 4 | 83.3 | 75.2-89.3 |  | 61 | 41 | 10 | 10 | 75.4 | 66.6-82.5 |
|  | PY-CFP LLIN | 109 | 74 | 26 | 9 | 79.8 | 73.7-84.8 |  | 65 | 45 | 15 | 5 | 80.8 | 72.7-86.9 |
| **Post2** | | |  |  |  |  |  |  |  |  |  |  |  |  |
| *An. coluzzii* | PY LLIN | 143 | 88 | 41 | 14 | 75.9 | 70.4-80.6 |  | 99 | 64 | 30 | 5 | 79.8 | 73.4-85 |
|  | PY-PPF LLIN | 120 | 76 | 38 | 6 | 79.2 | 73.4-84 |  | 117 | 72 | 37 | 8 | 77.3 | 71.3-82.4 |
|  | PY-CFP LLIN | 122 | 74 | 34 | 14 | 74.6 | 68.6-79.8 |  | 116 | 66 | 36 | 14 | 72.4 | 66.1-77.9 |
| *An. gambiae* s.s. | PY LLIN | 84 | 77 | 6 | 1 | 95.2 | 90.5-97.7 |  | 75 | 72 | 3 | 0 | 98 | 93.8-99.5 |
|  | PY-PPF LLIN | 88 | 82 | 6 | 0 | 96.6 | 92.4-98.6 |  | 60 | 59 | 1 | 0 | 99.2 | 94.7-99.9 |
|  | PY-CFP LLIN | 91 | 87 | 3 | 1 | 97.3 | 93.4-98.9 |  | 111 | 99 | 12 | 0 | 94.6 | 90.5-97.1 |

*An.: Anopheles*; N: number tested; PY LLIN: standard LLIN, LLIN treated with pyrethroid only; PY-CFP LLIN: LLIN bi-treated with pyrethroid-chlorfenapyr; PY-PPF LLIN: LLIN bi-treated with pyrethroid-pyriproxyfen; Post1: 1st year post-intervention; Post2: 2nd year post-intervention, CI: confidence interval
