# Supplementary material for: Impact of dual active ingredients long-lasting insecticidal nets on the genetic structure of insecticide resistant populations of Anopheles gambiae in Southern Benin
Source: Malar J. 2025 Mar 4;24:72. doi: 10.1186/s12936-025-05308-7 (PMC11877869; doi:10.1186/s12936-025-05308-7)
Supplement: Supplementary file 3 — Additional file 3: Table S3. Genotypic and allelic frequenciesof G119S and HWE test outdoor of Anopheles gambiae s.s. and Anopheles coluzzii populations. An.: Anopheles; N: number tested; PY LLIN: standard LLIN, LLIN treated with pyrethroid only; PY-CFP LLIN: LLIN bi-treated with pyrethroid-chlorfenapyr; PY-PPF LLIN: LLIN bi-treated with pyrethroid-pyriproxyfen; RR: homozygous resistant; RS: heterozygous resistant; SS: homozygous susceptible; p value: p value for Hardy–Weinberg Equilibrium; Post1: 1st year post-intervention; Post2: 2nd year post-intervention [file 12936_2025_5308_MOESM3_ESM.docx]

**Table S3:** Genotypic and allelic frequencies (Fr) of G119S and HWE test outdoor of *Anopheles gambiae* s.s. and *Anopheles coluzzii* populations

|  |  |  |  | **Outdoor** |  |  |  |
| --- | --- | --- | --- | --- | --- | --- | --- |
| **Period/ species** | **Study arms** | **N *An.*** | **Genotypic frequencies** | | | **Fr (G119S)** | **P-value (HWE)** |
|  |  |  | **RR (%)** | **RS (%)** | **SS (%)** |  |  |
| **Baseline** |  |  |  |  |  |  |  |
| *An. coluzzii* | PY LLIN | 125 | 0 (0) | 8 (6.4) | 117 (93.6) | 3.2 | 1 |
|  | PY-PPF LLIN | 123 | 0 (0) | 11 (8.9) | 112 (91.1) | 4.5 | 1 |
|  | PY-CFP LLIN | 126 | 0 (0) | 12 (9.5) | 114 (90.5) | 4.8 | 1 |
| *An. gambiae* s.s. | PY LLIN | 90 | 0 (0) | 6 (6.7) | 84 (93.3) | 3.3 | 1 |
|  | PY-PPF LLIN | 78 | 0 (0) | 4 (5.1) | 74 (94.9) | 2.6 | 1 |
|  | PY-CFP LLIN | 89 | 0 (0) | 5 (5.6) | 84 (94.4) | 2.8 | 1 |
| **Post1** | | |  |  |  |  |  |
| *An. coluzzii* | PY LLIN | 91 | 0 (0) | 1 (1.1) | 90 (98.9) | 0.6 | - |
|  | PY-PPF LLIN | 99 | 0 (0) | 4 (4.0) | 95 (96.0) | 2.0 | 1 |
|  | PY-CFP LLIN | 93 | 0 (0) | 6 (6.5) | 87 (93.6) | 3.2 | 1 |
| *An. gambiae s.s.* | PY LLIN | 60 | 0 (0) | 5 (8.3) | 55 (91.7) | 4.2 | 1 |
|  | PY-PPF LLIN | 61 | 0 (0) | 1 (1.6) | 60 (98.4) | 0.8 | 1 |
|  | PY-CFP LLIN | 65 | 0 (0) | 3 (4.6) | 62 (95.4) | 2.3 | 1 |
| **Post2** | | |  |  |  |  |  |
| *An. coluzzii* | PY LLIN | 99 | 0 (0) | 13 (11.2) | 103 (88.8) | 5.6 | 1 |
|  | PY-PPF LLIN | 116 | 0 (0) | 9 (9.1) | 90 (90.9) | 4.6 | 1 |
|  | PY-CFP LLIN | 117 | 0 (0) | 10 (8.6) | 107 (91.5) | 4.3 | 1 |
| *An. gambiae* s.s. | PY LLIN | 75 | 0 (0) | 11 (14.7) | 64 (85.3) | 7.3 | 1 |
|  | PY-PPF LLIN | 60 | 0 (0) | 10 (16.7) | 50 (83.3) | 8.3 | 1 |
|  | PY-CFP LLIN | 111 | 0 (0) | 18 (16.2) | 93 (83.8) | 8.1 | 1 |

*An.: Anopheles*; N: number tested; PY LLIN: standard LLIN, LLIN treated with pyrethroid only; PY-CFP LLIN: LLIN bi-treated with pyrethroid-chlorfenapyr; PY-PPF LLIN: LLIN bi-treated with pyrethroid-pyriproxyfen; RR: homozygous resistant; RS: heterozygous resistant; SS: homozygous susceptible; p value (HWE): p value for Hardy-Weinberg Equilibrium; Post1: 1st year post-intervention; Post2: 2nd year post-intervention
