# Supplementary material for: Impact of dual active ingredients long-lasting insecticidal nets on the genetic structure of insecticide resistant populations of Anopheles gambiae in Southern Benin
Source: Malar J. 2025 Mar 4;24:72. doi: 10.1186/s12936-025-05308-7 (PMC11877869; doi:10.1186/s12936-025-05308-7)
Supplement: Supplementary file 4 — Additional file 4: Table S4. Allele frequency of the G119S-ace-1 mutation in An. gambiae s.s. and An. coluzzii in the three study arms. An.: Anopheles; N: number tested; PY LLIN: standard LLIN, LLIN treated with pyrethroid only; PY-CFP LLIN: LLIN bi-treated with pyrethroid-chlorfenapyr; PY-PPF LLIN: LLIN bi-treated with pyrethroid-pyriproxyfen; Post1: 1st year post-intervention; Post2: 2nd year post-intervention, CI: confidence interval [file 12936_2025_5308_MOESM4_ESM.docx]

**Table S4 : Allele frequency of the G119S-*Ace-1* mutation in *An. gambiae* s.s. and *An. coluzzii* in the three study arms**

|  |  | **Indoor** | | | | | |  | **Outdoor** | | | | | |
| --- | --- | --- | --- | --- | --- | --- | --- | --- | --- | --- | --- | --- | --- | --- |
| **Period/Molecular species** | **Study arms** | **N *An*.** | **RR** | **RS** | **SS** | **Fr (*Ace-1*)** | **95% CI** |  | **N *An.*** | **RR** | **RS** | **SS** | **Fr (*Ace-1*)** | **95% CI** |
| **Baseline** |  |  |  |  |  |  |  |  |  |  |  |  |  |  |
| *An. coluzzii* | PY LLIN | 208 | 0 | 13 | 195 | 3.1 | 1.7-5.4 |  | 125 | 0 | 8 | 117 | 3.2 | 1.4-6.4 |
|  | PY-PPF LLIN | 218 | 0 | 14 | 204 | 3.2 | 1.8-5.5 |  | 123 | 0 | 11 | 112 | 4.5 | 2.3-8.1 |
|  | PY-CFP LLIN | 168 | 0 | 13 | 155 | 3.8 | 2.1-6.7 |  | 126 | 0 | 12 | 114 | 4.7 | 2.5-8.4 |
| *An. gambiae* s.s. | PY LLIN | 177 | 0 | 12 | 165 | 3.4 | 1.8-6 |  | 90 | 0 | 6 | 84 | 3.3 | 1.4-7.4 |
|  | PY-PPF LLIN | 174 | 0 | 13 | 161 | 3.7 | 2.1-6.5 |  | 78 | 0 | 4 | 74 | 2.6 | 0.8-6.8 |
|  | PY-CFP LLIN | 221 | 0 | 14 | 207 | 3.2 | 1.8-5.4 |  | 89 | 0 | 5 | 84 | 2.8 | 1-6.8 |
| **Post1** | | |  |  |  |  |  |  |  |  |  |  |  |  |
| *An. coluzzii* | PY LLIN | 167 | 0 | 6 | 161 | 1.8 | 0.7-4.1 |  | 91 | 0 | 1 | 90 | 0.5 | 0.03-3.5 |
|  | PY-PPF LLIN | 148 | 0 | 5 | 143 | 1.7 | 0.6-4.1 |  | 99 | 0 | 4 | 95 | 2 | 0.6-5.4 |
|  | PY-CFP LLIN | 140 | 0 | 1 | 139 | 0.3 | 0.01-2.3 |  | 93 | 0 | 6 | 87 | 3.2 | 1.3-7.2 |
| *An. gambiae* s.s. | PY LLIN | 124 | 0 | 1 | 123 | 0.4 | 0.02-2.6 |  | 60 | 0 | 5 | 55 | 4.2 | 1.5-9.9 |
|  | PY-PPF LLIN | 60 | 0 | 2 | 58 | 1.7 | 0.3-6.5 |  | 61 | 0 | 1 | 60 | 0.8 | 0.04-5.2 |
|  | PY-CFP LLIN | 109 | 0 | 1 | 108 | 0.4 | 0.02-2.9 |  | 65 | 0 | 3 | 62 | 2.3 | 0.6-7.1 |
| **Post2** | | |  |  |  |  |  |  |  |  |  |  |  |  |
| *An. coluzzii* | PY LLIN | 143 | 0 | 14 | 129 | 4.9 | 2.8-8.3 |  | 116 | 0 | 13 | 103 | 5.6 | 3.1-9.6 |
|  | PY-PPF LLIN | 120 | 0 | 16 | 104 | 6.7 | 3.9-10.8 |  | 99 | 0 | 9 | 90 | 4.5 | 2.2-8.7 |
|  | PY-CFP LLIN | 122 | 0 | 10 | 112 | 4.1 | 2.1-7.6 |  | 117 | 0 | 10 | 107 | 4.3 | 2.2-7.9 |
| *An. gambiae* s.s. | PY LLIN | 84 | 0 | 9 | 75 | 5.4 | 2.6-10.2 |  | 75 | 0 | 11 | 64 | 7.33 | 3.9-13.1 |
|  | PY-PPF LLIN | 88 | 0 | 12 | 76 | 6.8 | 3.7-11.9 |  | 60 | 0 | 10 | 50 | 8.3 | 4.3-15.2 |
|  | PY-CFP LLIN | 91 | 0 | 12 | 79 | 6.6 | 3.6-11.5 |  | 111 | 0 | 18 | 93 | 8.1 | 5-12.7 |

*An.: Anopheles*; N: number tested; PY LLIN: standard LLIN, LLIN treated with pyrethroid only; PY-CFP LLIN: LLIN bi-treated with pyrethroid-chlorfenapyr; PY-PPF LLIN: LLIN bi-treated with pyrethroid-pyriproxyfen; Post1: 1st year post-intervention; Post2: 2nd year post-intervention, CI: confidence interval
