# Supplementary material for: Impact of dual active ingredients long-lasting insecticidal nets on the genetic structure of insecticide resistant populations of Anopheles gambiae in Southern Benin
Source: Malar J. 2025 Mar 4;24:72. doi: 10.1186/s12936-025-05308-7 (PMC11877869; doi:10.1186/s12936-025-05308-7)
Supplement: Supplementary file 5 — Additional file 5: Table S5. Expected heterozygousand observed heterozygouswithin the locus L1014F-vgsc-kdr in An. gambiae s.s. and An. coluzzii species. An.: Anopheles; N: number tested; PY LLIN: standard LLIN, LLIN treated with pyrethroid only; PY-CFP LLIN: LLIN bi-treated with pyrethroid-chlorfenapyr; PY-PPF LLIN: LLIN bi-treated with pyrethroid-pyriproxyfen; Post1: 1st year post-intervention; Post2: 2nd year post-intervention. [file 12936_2025_5308_MOESM5_ESM.docx]

**Table S5**: Expected heterozygous (*He*) and observed heterozygous (*Ho*) within the locus L1014F *vgsc-kdr* in *An. gambiae* s.s. and *An. coluzzii* species

|  |  | ***An. coluzzii* (L1014F)** | | |  | ***An. gambiae s.s.* (L1014F)** | | |
| --- | --- | --- | --- | --- | --- | --- | --- | --- |
| **Location/**  **LLINs** | **Period** | **N *An*.** | ***He*** | ***Ho*** |  | **N *An.*** | ***He*** | ***Ho*** |
| **Indoor** |  |  |  |  |  |  |  |  |
| PY LLIN | Baseline | 208 | 0.293 | 0.250 |  | 177 | 0.141 | 0.13 |
|  | Post1 | 167 | 0.299 | 0.234 |  | 124 | 0.363 | 0.26 |
|  | Post2 | 143 | 0.364 | 0.287 |  | 84 | 0.095 | 0.11 |
|  | Baseline | 218 | 0.26 | 0.225 |  | 174 | 0.138 | 0.15 |
| PY-PPF LLIN | Post1 | 148 | 0.361 | 0.257 |  | 60 | 0.267 | 0.2 |
|  | Post2 | 120 | 0.325 | 0.308 |  | 88 | 0.125 | 0.14 |
| PY-CFP LLIN | Baseline | 168 | 0.229 | 0.190 |  | 221 | 0.190 | 0.16 |
|  | Post1 | 140 | 0.321 | 0.229 |  | 109 | 0.321 | 0.240 |
|  | Post2 | 122 | 0.377 | 0.279 |  | 91 | 0.121 | 0.130 |
| **Outdoor** |  |  |  |  |  |  |  |  |
| PY-LLIN | Baseline | 125 | 0.218 | 0.184 |  | 90 | 0.257 | 0.230 |
|  | Post1 | 91 | 0.277 | 0.220 |  | 60 | 0.222 | 0.150 |
|  | Post2 | 99 | 0.324 | 0.303 |  | 75 | 0.039 | 0.040 |
| PY-PPF LLIN | Baseline | 123 | 0.295 | 0.228 |  | 78 | 0.215 | 0.19 |
|  | Post1 | 99 | 0.353 | 0.192 |  | 61 | 0.374 | 0.16 |
|  | Post2 | 117 | 0.352 | 0.316 |  | 60 | 0.017 | 0.02 |
| PY-CFP LLIN | Baseline | 126 | 0.278 | 0.222 |  | 89 | 0.226 | 0.21 |
|  | Post1 | 93 | 0.363 | 0.258 |  | 65 | 0.314 | 0.23 |
|  | Post2 | 116 | 0.401 | 0.310 |  | 111 | 0.103 | 0.110 |

*An.: Anopheles*; N: number tested; PY LLIN: standard LLIN, LLIN treated with pyrethroid only; PY-CFP LLIN: LLIN bi-treated with pyrethroid-chlorfenapyr; PY-PPF LLIN: LLIN bi-treated with pyrethroid-pyriproxyfen; Post1: 1st year post-intervention; Post2: 2nd year post-intervention.
