# Supplementary material for: Impact of dual active ingredients long-lasting insecticidal nets on the genetic structure of insecticide resistant populations of Anopheles gambiae in Southern Benin
Source: Malar J. 2025 Mar 4;24:72. doi: 10.1186/s12936-025-05308-7 (PMC11877869; doi:10.1186/s12936-025-05308-7)
Supplement: Supplementary file 6 — Additional file 6: Table S6. Expected heterozygousand observed heterozygouswithin the locus Ace-1 in An. gambiae s.s and An. coluzzii species. An.: Anopheles gambiae s.l.; N: number tested; PY LLIN: standard LLIN, LLIN treated with pyrethroid only; PY-CFP LLIN: LLIN bi-treated with pyrethroid-chlorfenapyr; PY-PPF LLIN: LLIN bi-treated with pyrethroid-pyriproxyfen; Post1: 1st year post-intervention; Post2: 2nd year post-intervention. [file 12936_2025_5308_MOESM6_ESM.docx]

**Table S6:** Expected heterozygous (*He*) and observed heterozygous (*Ho*) within the locus *Ace*-*1* in *An. gambiae* s.s and *An. coluzzii* species

|  |  | ***An. coluzzii (*G119S*)*** | | |  | ***An. gambiae s.s.* (G119S)** | | |
| --- | --- | --- | --- | --- | --- | --- | --- | --- |
| **Location**  **/ LLINs** | **Periods** | **N *An.*** | ***He*** | ***Ho*** |  | **N *An.*** | ***He*** | ***Ho*** |
| **Indoor** |  |  |  |  |  |  |  |  |
| PY LLIN | Baseline | 208 | 0.061 | 0.063 |  | 177 | 0.066 | 0.068 |
|  | Post1 | 167 | 0.035 | 0.036 |  | 124 | 0.008 | 0.008 |
|  | Post2 | 143 | 0.093 | 0.098 |  | 84 | 0.102 | 0.107 |
|  | Baseline | 218 | 0.062 | 0.064 |  | 174 | 0.078 | 0.08 |
| PY-PPF LLIN | Post1 | 148 | 0.033 | 0.034 |  | 60 | 0.032 | 0.033 |
|  | Post2 | 120 | 0.125 | 0.133 |  | 88 | 0.127 | 0.136 |
| PY-CFP LLIN | Baseline | 168 | 0.074 | 0.077 |  | 221 | 0.062 | 0.063 |
|  | Post1 | 140 | 0.007 | 0.007 |  | 109 | 0.009 | 0.009 |
|  | Post2 | 122 | 0.078 | 0.082 |  | 91 | 0.124 | 0.132 |
| **Outdoor** |  |  |  |  |  |  |  |  |
| PY LLIN | Baseline | 125 | 0.062 | 0.064 |  | 90 | 0.064 | 0.067 |
|  | Post1 | 91 | 0.011 | 0.011 |  | 60 | 0.080 | 0.083 |
|  | Post2 | 116 | 0.106 | 0.112 |  | 75 | 0.137 | 0.147 |
| PY-PPF LLIN | Baseline | 123 | 0.086 | 0.089 |  | 78 | 0.05 | 0.051 |
|  | Post1 | 99 | 0.039 | 0.04 |  | 61 | 0.031 | 0.033 |
|  | Post2 | 99 | 0.086 | 0.091 |  | 60 | 0.153 | 0.167 |
| PY-CFP LLIN | Baseline | 126 | 0.084 | 0.087 |  | 89 | 0.055 | 0.056 |
|  | Post1 | 93 | 0.062 | 0.065 |  | 65 | 0.045 | 0.046 |
|  | Post2 | 117 | 0.082 | 0.085 |  | 111 | 0.15 | 0.162 |

*An.: Anopheles*; N: number tested; PY LLIN: standard LLIN, LLIN treated with pyrethroid only; PY-CFP LLIN: LLIN bi-treated with pyrethroid-chlorfenapyr; PY-PPF LLIN: LLIN bi-treated with pyrethroid-pyriproxyfen; Post1: 1st year post-intervention; Post2: 2nd year post-intervention.
